# Supplementary material for: Demethylation C–C coupling reaction facilitated by the repulsive Coulomb force between two cations
Source: Nat Commun. 2024 Jul 13;15:5881. doi: 10.1038/s41467-024-49946-y (PMC11245495; doi:10.1038/s41467-024-49946-y)
Supplement: Supplementary file 1 — Supplementary Information [file 41467_2024_49946_MOESM1_ESM.pdf]

Supplementary Information

Demethylation C–C coupling reaction facilitated by the repulsive Coulomb force between two cations

Xiaoping Zhang[1], Keke Huang[2], Yanlin Fu[3], Ni Zhang[4], Xianglei Kong[5], Yuanyuan Cheng[1], Mingyu Zheng[1], Yihao Cheng[1], Tenggao Zhu[4], Bina Fu[3]*, Shouhua Feng[2]*, Huanwen Chen[1,4]*

[1]Jiangxi Key Laboratory for Mass Spectrometry and Instrumentation, East China University of Technology, Nanchang, 330013, P. R. China.

[2]State Key Laboratory of Inorganic Synthesis and Preparative Chemistry, Jilin University, Changchun, 130012, P. R. China.

[3]State Key Laboratory of Molecular Reaction Dynamics, Center for Theoretical and Computational Chemistry, Dalian Institution of Chemical Physics, Chinese Academy of Sciences, Dalian, 116023, P. R. China.

[4]School of Pharmacy, Jiangxi University of Chinese Medicine, Nanchang, 330004, P. R. China.

[5]State Key Laboratory of Elemento-organic Chemistry, College of Chemistry, Nankai University, Tianjin, 300071, P. R. China.

*Corresponding authors.

Email: bina@dicp.ac.cn, shfeng@jlu.edu.cn, and chw8868@gmail.com

These authors contributed equally: Xiaoping Zhang, Keke Huang, Yanlin Fu.

This file includes:

- Supplementary Note 1
- Supplementary Note 2
- Supplementary Figure 1 to 31
- Supplementary Tables 1 to 3
- The imaginary vibrational frequency of TS1 and TS2
- Supplementary references

Table of Contents

1. Supplementary Note 1: Electric field value in interface…………………………………..4-5

2. Supplementary Note 2: Yield calculations……………………………….. ……………6-7

Supplementary Figure 1……………………………………………………………………….8

Supplementary Figure 2………………………………………………………………………9

Supplementary Figure 3……………………………………………………………………10-11

Supplementary Figure 4………………………………………………………………………12

Supplementary Figure 5……………………………………………………………………13-14

Supplementary Figure 6………………………………………………………………………15

Supplementary Figure 7………………………………………………………………………16

Supplementary Figure 8………………………………………………………………………17

Supplementary Figure 9………………………………………………………………………18

Supplementary Figure 10……………………………………………………………………..19

Supplementary Figure 11……………………………………………………………………..20

Supplementary Figure 12……………………………………………………………………..21

Supplementary Figure 13…………………………………………………………………….22-23

Supplementary Figure 14……………………………………………………………………..24

Supplementary Figure 15……………………………………………………………………..25

Supplementary Figure 16……………………………………………………………………..26

Supplementary Figure 17……………………………………………………………………..27

Supplementary Figure 18……………………………………………………………………..28

Supplementary Figure 19……………………………………………………………………..29

Supplementary Figure 20……………………………………………………………………..30

Supplementary Figure 21……………………………………………………………………..31

Supplementary Figure 22……………………………………………………………………..32

Supplementary Figure 23……………………………………………………………………..33

Supplementary Figure 24……………………………………………………………………..34

Supplementary Figure 25……………………………………………………………………..35

Supplementary Figure 26……………………………………………………………………..36

Supplementary Figure 27……………………………………………………………………..37

Supplementary Figure 28…………………………………………………………………….38-39

Supplementary Figure 29……………………………………………………………………..40
Supplementary Figure 30…………………………………………………………………….41-42
Supplementary Figure 31……………………………………………………………………..43
Supplementary Table 1…………………………………………………………………44-47
Supplementary Table 2……………………………………………………………………48
Supplementary Table 3…………………………………………………………………49-50
The imaginary vibrational frequency of TS1 and TS2……………………………………51-52
Supplementary References………………………………………………………………53

1. Supplementary Note 1: Electric field value in interface

The value quoted from Zare and coworkers (10^9 V m^{-1}) is electric field present in neutral droplets. However, the droplets in our work are in charged. The voltage supplied to the electrosrapy plume and surface is not only used to generate sodiated cations, but also energetically play a role in formation of carbon chain elongation (CCE) products. While an electric field has been implicated at the water interface, there has been no direct measurement in aqueous microdroplets, largely due to the lack of proper measurements tools. Zare and coworkers employed the newly developed stimulated Raman excited fluorescence microscopy to measure the electric field at the water-oil interface of microdroplets, in which the droplets are fixed [1]. As determined by vibrational Stark effect of a nitril-bearing fluorescent probe, the strength of the electric field is found to be on the order of 10^9 V m^{-1}. However, in our work, the droplets are charged, which can be attracted by an electric field, and is not easily fixed, so it is difficult to measure the electric field value by using the reference method reported by Zare and coworkers. According to the research literatures [1-4], both the theoretical calculation and the experimental measurement of the electric field at the neutral droplet interface are of the order of 10^9 V m^{-1}. For example, Cendagorta and Ichiye [3] reported the formation of water-vapor interface potential as high as 0.4 V with the corresponding electric field at 0.8×10^9 V m^{-1}, which originates from the aligned orientation of water dipoles at the interface. Leung [4] used density functional theory to estimate the water surface potential and the interfacial electric field to be approximately +3.63 V and 10^9 V m^{-1}. According to the reference results, the water surface potential is related to the interfacial electric field. In our work, due to the application of a voltage of several kilovolts, a large amount of charge was accumulated on the surface of the water droplets, thus the interfacial electric field could be greater than 10^9 V m^{-1}. This high electric field plays a key role in the formation of CCE products energetically. At the present stage, we

could not calculate the electric field value of the surface of the charged droplets by theoretical or experimental methods. However, it is certain that this value is greater than the electric field value on the surface of the neutral droplets. In our future work plan, we will try to calculate the electric field value at the air water interface in experiments.

2. Supplementary Note 2: Yield calculations

The method for yield calculation in this work was derived from the rough estimates of the yields of the microdroplet synthesis reactions reported by Cooks [5,6] group and Zare [7,8] group, which was obtained by measuring the conversion ratio: the ratio of the intensity between the product (P) and the sum of the intensities of the reactant (R), and product (P), e.g., [P]/([R]+[P]). In this work, the yields were determined as the ratio of the sum of the intensity of C−C coupling product ions to the sum of the intensity of reactant ions and the intensity of C−C coupling product ions.

For the gas-phase reaction in the ion trap, taking CCE reaction between two sodiated acetone cations as an example, the detailed calculation process is outlined as follows: the yield of CCE reaction for sodiated acetone was determined by calculating the ratio of the intensity of CCE product ions at *m/z* 147 [CH_3C^+(ONa)-C(ONa)$(CH_3)_2$] to the sum of the intensity of reactant ions at *m/z* 81 [CH_3C^+(ONa)CH_3] and C−C coupling product ions at *m/z* 147 (Fig. 4a). The signal intensity of *m/z* 81 and *m/z* 147 were 8.5 and 2.5, respectively. Consequently, the yield for CCE reaction between two sodiated acetone cations in the gas-phase was calculated as approximately 25%.

For the liquid phase reaction in the Fig. 2a, taking CCE reaction between protonated/sodiated acetone as an example, the detailed calculation process is outlined as follows: the yield of CCE reaction for protonated/sodiated acetone was determined by calculating the ratio of the sum of the intensity of CCE product ions at *m/z* 103 [CH_3C^+(OH)-C(OH)$(CH_3)_2$], *m/z* 105 [CH_3CH(OH)-C(OH)$(CH_3)_2$ + H]$^+$, *m/z* 147 [CH_3C^+(ONa)-C(ONa)$(CH_3)_2$], *m/z* 149 [CH_3CH(ONa)-C(ONa)$(CH_3)_2$ + H)$^+$], *m/z* 163 [CH_3(CH_3COO)C(OH)-C(OH)$(CH_3)_2$ + H]$^+$, and *m/z* 207 [CH_3(CH_3COO)C(ONa)-C(ONa)$(CH_3)_2$) + H]$^+$ to the sum of the intensity of reactant ions at *m/z* 59 [CH_3C^+(OH)CH_3], *m/z* 81 [CH_3C^+(ONa)CH_3] and CCE product ions at *m/z* 103, *m/z* 105, *m/z*

147, *m/z* 149, *m/z* 163, and *m/z* 207 (Supplementary Fig. 5a). The signal intensity of *m/z* 59, *m/z* 81, *m/z* 103, *m/z* 105, *m/z* 147, *m/z* 149, *m/z* 163, and *m/z* 207 were 7.38×10^5, 1.85×10^5, 1.72×10^4, 1.71×10^4, 1.92×10^4, 1.99×10^4, 1.32×10^4, 7.00×10^4, respectively. Consequently, CCE yield for protonated/sodiated acetone was calculated as approximately 14%.

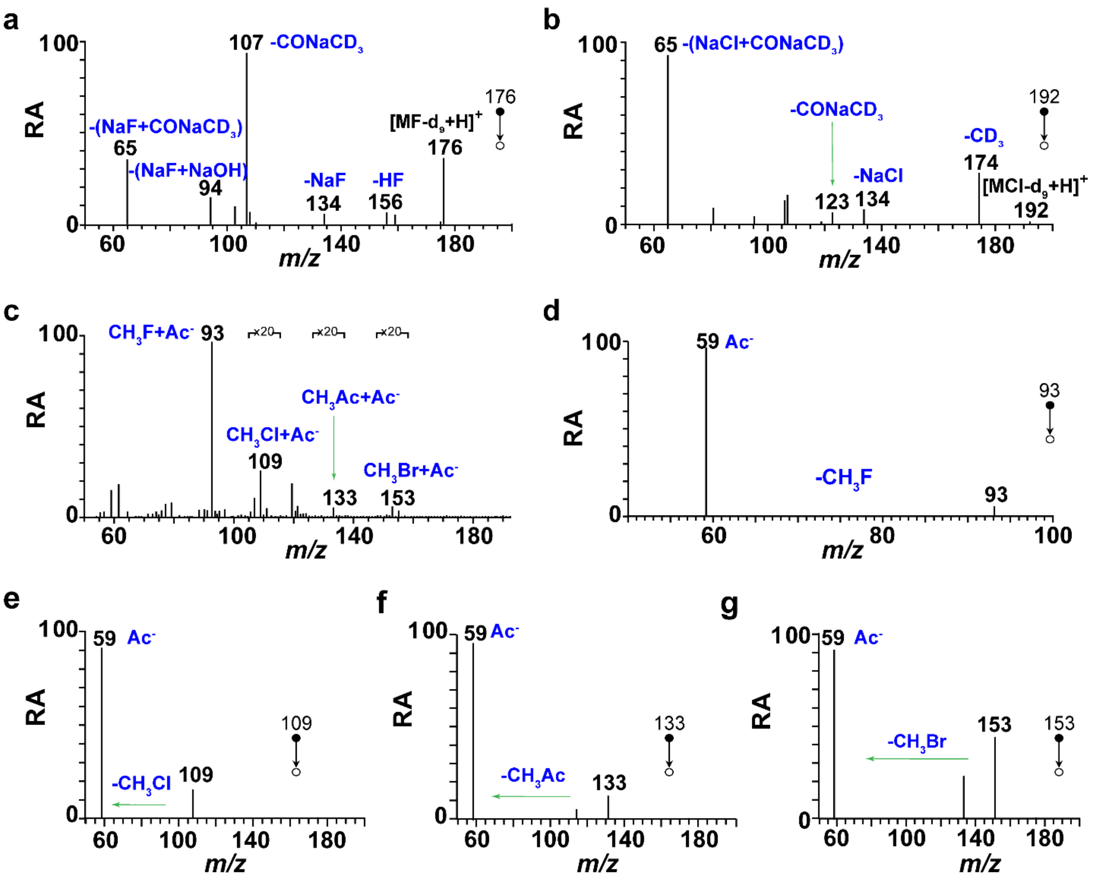

Supplementary Figure 1. The tandem mass spectra of the demethylation C–C coupling products formed between two sodiated acetone-d6 cations and the capture of CH_3^+ during demethylation C–C coupling reaction between two sodicated acetone cations. a Tandem mass spectrum of *m/z* 176 ($MF\text{-}d_9 + H)^+$ in Fig. 2d. **b** Tandem mass spectrum of *m/z* 192 ($MCl\text{-}d_9 + H)^+$ in Fig. 2d. **c** Full mass spectrum of methyl cation captured by anions such as F^-, Cl^-, Br^- and CH_3COO^-. **d** Tandem mass spectrum of *m/z* 93 in Supplementary Fig. 2c. **e** Tandem mass spectrum of *m/z* 109 in Supplementary Fig. 1c. **f** Tandem mass spectrum of *m/z* 133 in Supplementary Fig. 1c. **g** Tandem mass spectrum of *m/z* 153 in Supplementary Fig. 1c. RA denotes Relative Abundance. **M**=$R_2C(OH/Na)\text{-}C(OH/Na)R_4R_3$, R_1, R_2, and R_3 all denote $-CH_3$ group.

The mass spectral data collected under the negative ion detection mode by electrospray of the liquid solution after the CCE reaction. The common product of CCE reactions, CH_3^+ ion was efficiently captured by anions such as F^-, Cl^-, Br^- CH_3COO^- to form CH_3F, CH_3Cl, CH_3Br and CH_3COOCH_3 respectively in the solution, which then generated the corresponding signals at *m/z* 93, *m/z* 109, *m/z* 153 and *m/z* 133 by forming the (CH_3X+Ac^-) adducts as detected in the negative ion detection mode.

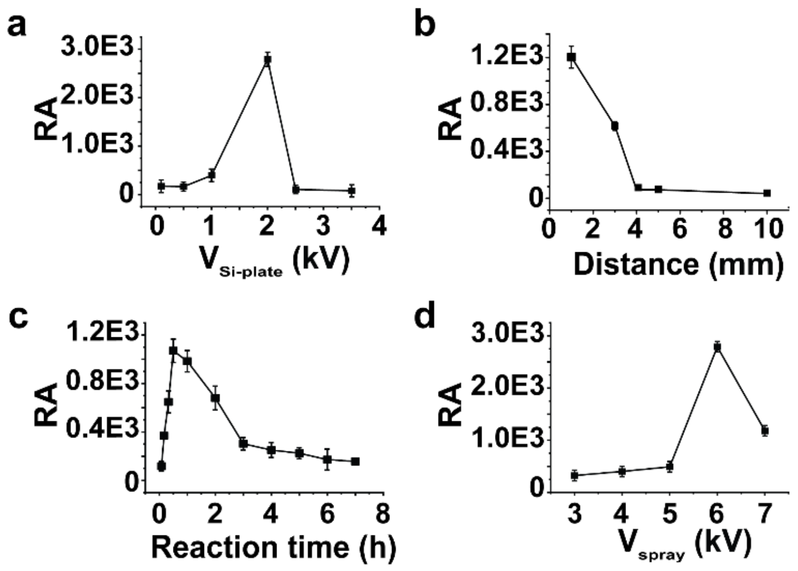

Supplementary Figure 2. Optimization of the experimental conditions for the demethylation C–C coupling reaction. a The voltage of the coated silicon electrode. **b** The distance from the spray tip to the surface of the solution in which the coated silicon electrode was placed. **c** The reaction time. **d** The voltage of spray. RA denotes Relative Abundance.

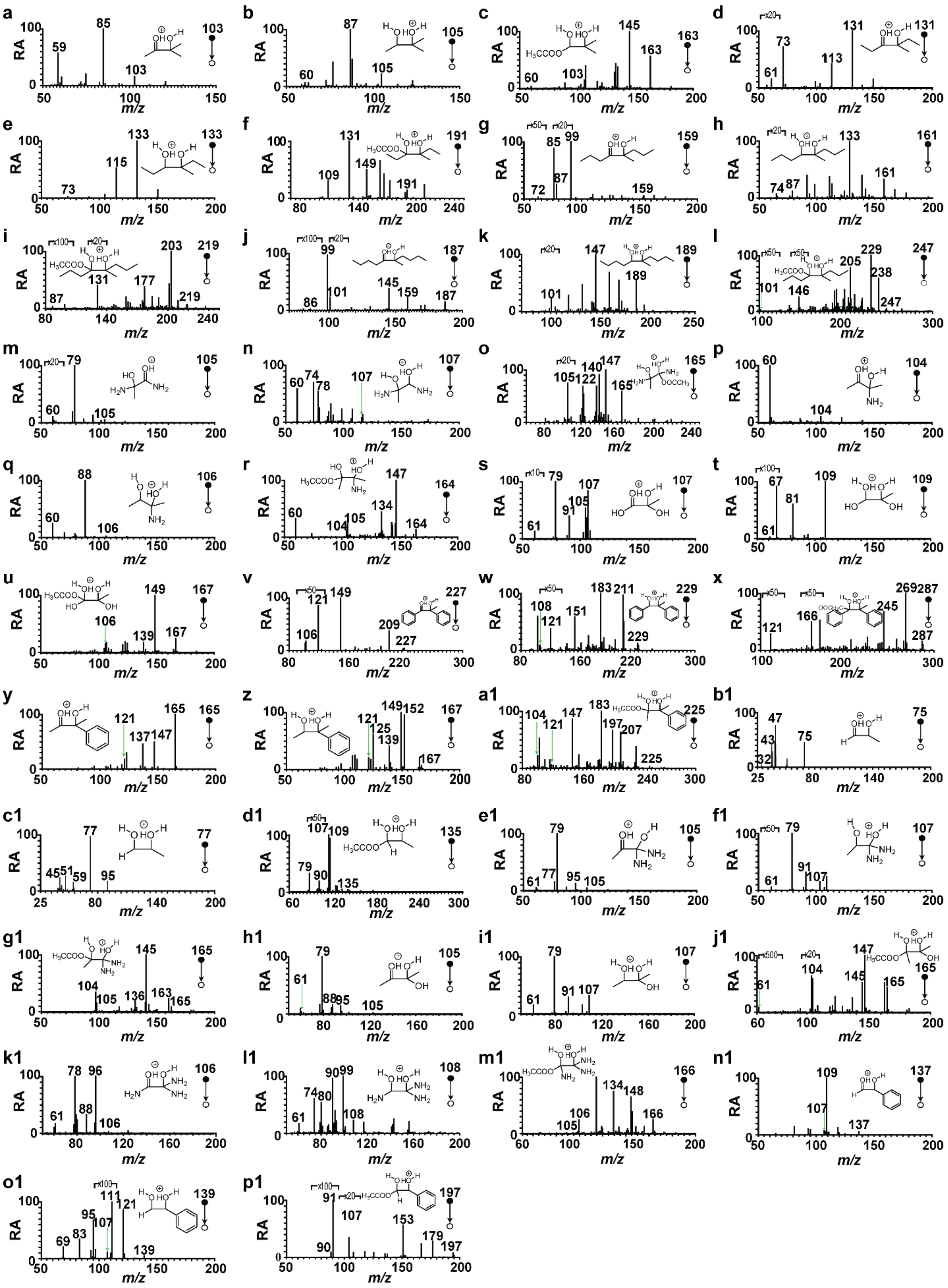

Supplementary Figure 3. Tandem mass spectra of the products produced via demethylation C–C coupling reactions conducted using the experimental setup shown in Fig. 2a with series of chemicals containing different functional groups. Protonated acetone and protonated acetone (**a-c**). Protonated butanone and protonated butanone (**d-f**). Protonated 2-pentanone and protonated 2-pentanone (**g-i**). Protonated 2-hexanone and protonated 2-hexanone (**j-l**). Protonated acetamide and protonated acetamide (**m-r**). Protonated acetic acid and protonated acetic acid (**s-u**). Protonated acetophenone and protonated acetophenone (**v-a1**). Protonated acetaldehyde and protonated acetaldehyde (**b1-d1**). Protonated acetone and protonated urea (**e1-g1**). Protonated acetone and protonated acetic acid (**h1-j1**). Protonated urea and protonated urea (**k1-m1**). Protonated benzaldehyde and protonated benzaldehyde (**n1-p1**). RA denotes Relative Abundance.

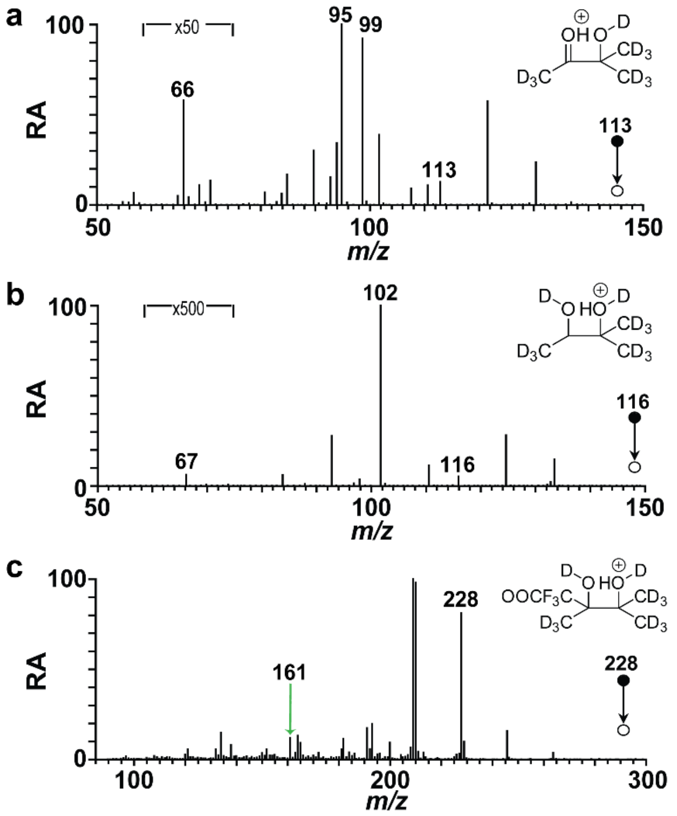

Supplementary Figure 4. Tandem mass spectra of the products of acetone-d$_6$ produced via demethylation C–C coupling reaction conducted using the experimental setup shown in Fig. 2 with series of chemicals containing different functional groups. a Tandem mass spectrum of CCE product (*m/z* 113) of protonated acetone-d$_6$ and protonated acetone-d$_6$. **b** Tandem mass spectrum of CCE product (*m/z* 116) of protonated acetone-d$_6$ and protonated acetone-d$_6$. **c** Tandem mass spectrum of CCE product (*m/z* 228) of protonated acetone-d$_6$ and protonated acetone-d$_6$. RA denotes Relative Abundance. CCE: carbon chain elongation.

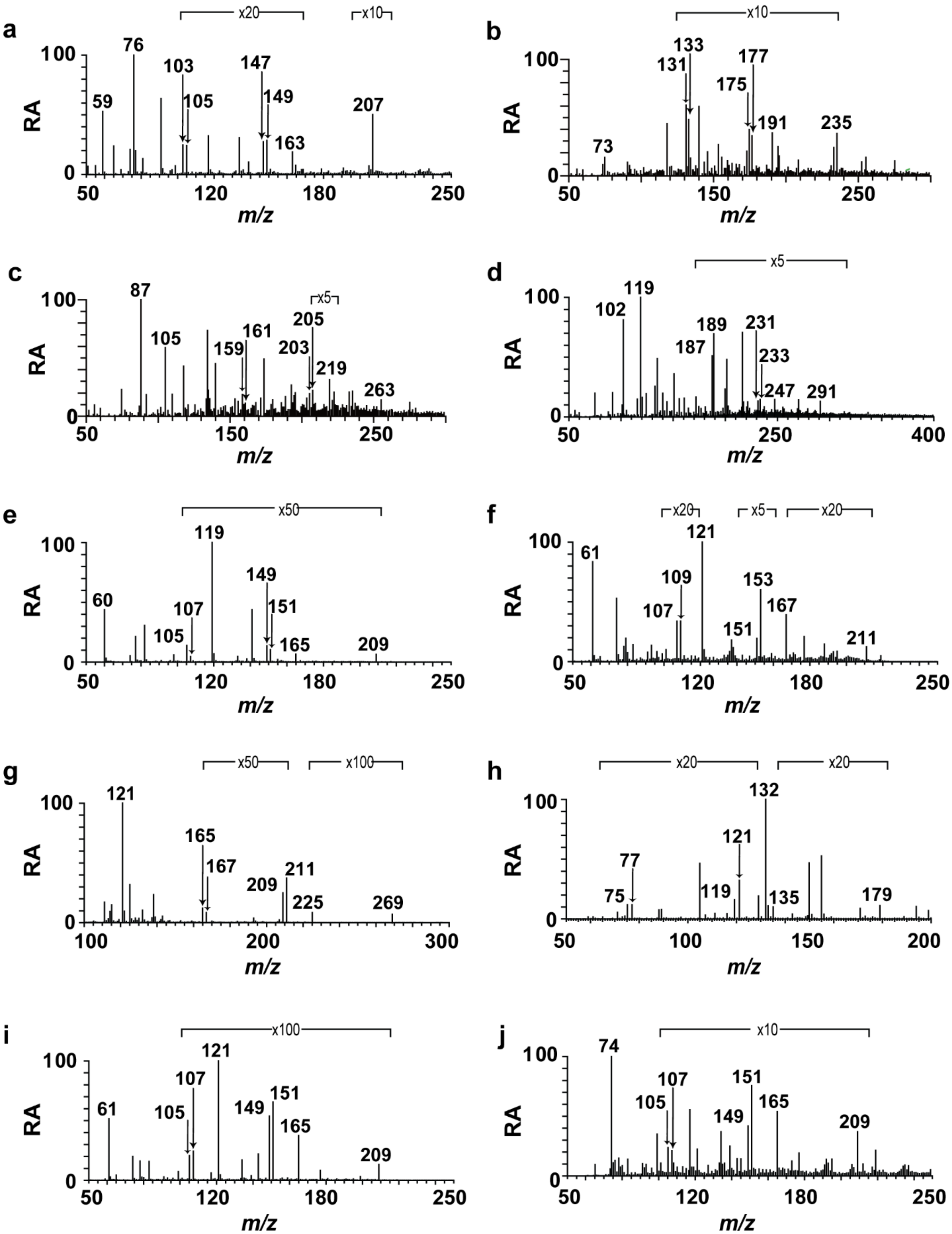
a
x20
x10
RA
100
0
76
59
103
105
147
149
163
207
50
120
180
250
m/z
b
x10
RA
100
0
133
131
177
175
191
235
73
50
150
250
m/z
c
x5
RA
100
0
87
105
159
161
205
203
219
263
50
150
250
m/z
d
x5
RA
100
0
119
102
189
187
231
233
247
291
50
250
400
m/z
e
x50
RA
100
0
119
60
107
105
149
151
165
209
50
120
180
250
m/z
f
x20
x5
x20
RA
100
0
61
121
109
107
153
151
167
211
50
120
180
250
m/z
g
x50
x100
RA
100
0
121
165
167
209
211
225
269
100
200
300
m/z
h
x20
x20
RA
100
0
132
121
77
75
119
135
179
50
100
150
200
m/z
i
x100
RA
100
0
121
107
61
105
149
151
165
209
50
120
180
250
m/z
j
x10
RA
100
0
74
107
105
151
165
149
209
50
120
180
250
m/z

Supplementary Figure 5. Mass spectra of demethylation C–C coupling reactions conducted using the experimental setup shown in Fig. 2a with protonated or sodiated compounds. a Mass spectrum of CCE product of protonated/sodiated acetone and protonated/sodiated acetone. **b** Mass spectrum of CCE product of protonated/sodiated butanone and protonated/sodiated butanone. **c** Mass spectrum of CCE product of protonated/sodiated 2-pentanone and protonated/sodiated 2-pentanone. **d** Mass spectrum of CCE product of protonated/sodiated 2-hexanone and protonated/sodiated 2-hexanone. **e** Mass spectrum of CCE product of protonated/sodiated acetamide and protonated/sodiated acetamide. **f** Mass spectrum of CCE product of protonated/sodiated acetic acid and protonated/sodiated acetic acid. **g** Mass spectrum of CCE product of protonated/sodiated acetophenone and protonated/sodiated acetophenone. **h** Mass spectrum of CCE product of protonated/sodiated acetaldehyde and protonated/sodiated acetaldehyde. **i** Mass spectrum of CCE product of protonated/sodiated acetone and protonated/sodiated urea. **j** Mass spectrum of CCE product of protonated acetone/sodiated and protonated/sodiated acetic acid. The red peaks are the coupling products. RA denotes Relative Abundance. CCE: carbon chain elongation.

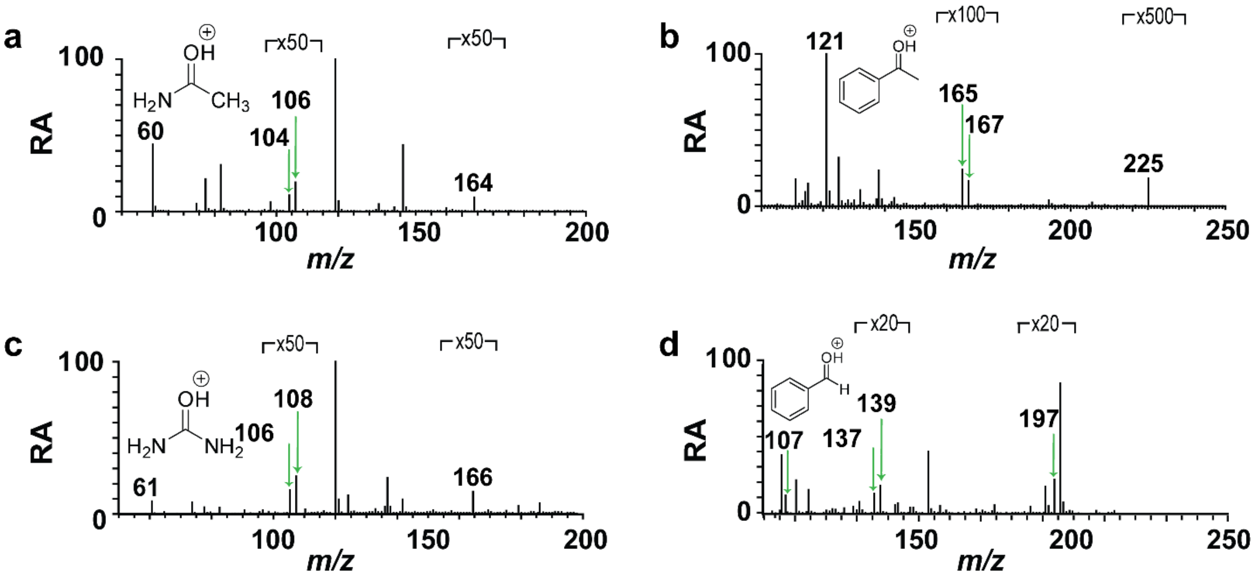

Supplementary Fiure 6. Mass spectra of deamination/dephenylation C–C coupling reaction conducted using the experimental setup shown in Fig. 2a. a Mass spectrum of CCE product of protonated acetamide and protonated acetamide. **b** Mass spectrum of CCE product of protonated acetophenone and protonated acetophenone. **c** Mass spectrum of CCE product of protonated urea and protonated urea. **d** Mass spectrum of CCE product of protonated acetaldehyde and protonated acetaldehyde. The red peaks are the resultants of the CCE reactions and electronically neutralized products. i.e., *m/z* 104, *m/z* 106 and *m/z* 164 in Supplementary Fig. 6a represents M, protonated MH and protonated MCH_3COO ($M = CH_3C^+(OH)–C(OH)CH_3NH_2$). The signal at *m/z* 165, *m/z* 167 and *m/z* 225 in Supplementary Fig. 6b represents M, protonated MH and protonated MCH_3COO ($M = CH_3C^+(OH)–C(OH)CH_3C_6H_5$). The signals at *m/z* 106, *m/z* 108 and *m/z* 166 in Supplementary Fig. 6c represents M, protonated MH and protonated MCH_3COO ($M = NH_2C^+(OH)–C(OH)NH_2NH_2$). The signal at *m/z* 137, *m/z* 139 and *m/z* 197 in Supplementary Fig. 6d represents M, protonated MH and protonated MCH_3COO ($M = HC^+(OH)–C(OH)HC_6H_5$). RA denotes Relative Abundance. CCE: carbon chain elongation.

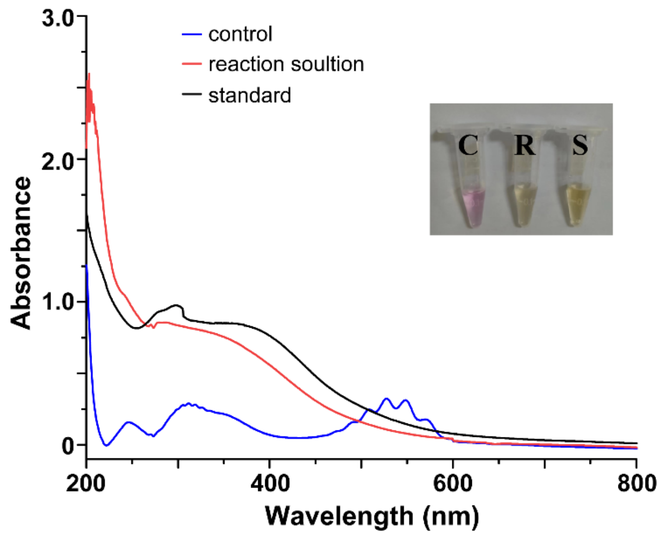

Supplementary Figure 7. Characterization of reaction (R) product by UV-Vis spectroscopy. Control (C): $KMnO_4$; Standard (S): 2-methylbutane-2,3-diol.

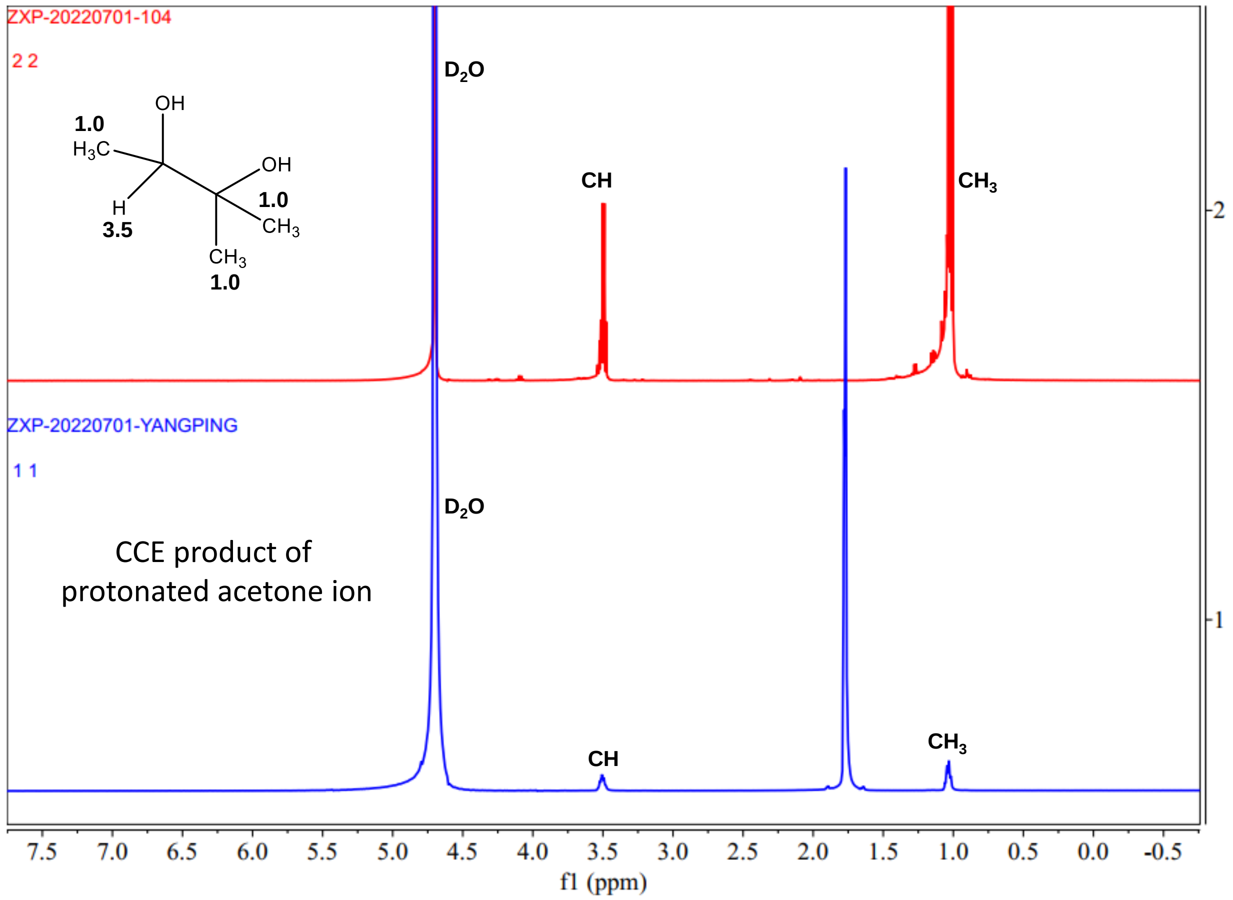

Supplementary Figure 8. ^{1}H nuclear magnetic resonance (500 MHz, D_2O) spectrum of 2-methylbutane-2,3-diol and carbon chain elongation (CCE) product of protonated acetone ion.

Using the device shown in Fig. 2a with properly programmed electric field, the demethylation C−C coupling experiment was performed with acetone aqueous solution as the reactant. After being worked for about 50 hours under the experimental conditions, the reactor produced resultant mixture, which was mainly consistent of the final product, water and acetone. After purification including salting-out extraction and evaporation, a colorless liquid as the final product of the demethylation C−C coupling experiment using protonated acetone was obtained for nuclear magnetic resonance analysis. As the result, the characteristic 1H signals of the product (the blue line) are in agreement with those signals recorded using the authentic 2-methylbutane-2,3-diol (the red line) in the nuclear magnetic resonance spectrum.

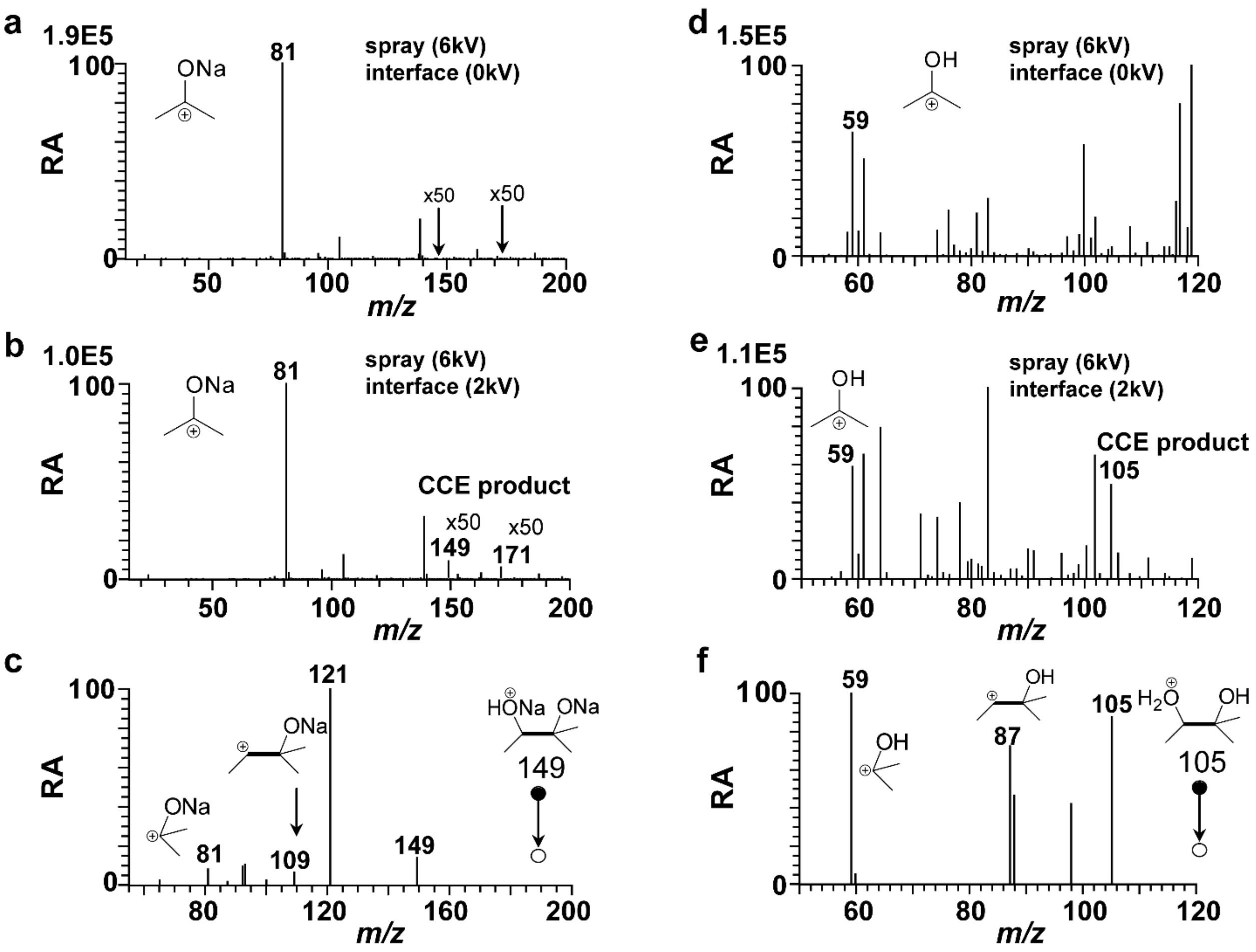

Supplementary Figure 9. Control experiments for CCE products formation by setup in Fig. 2a. a Mass spectrum of CCE reaction of two sodiated acetone cations when the spray charged while the interface not charged. **b** Mass spectrum of CCE reaction of two sodiated acetone cations when the spray and interface both charged. **c** Tandem mass spectrum of CCE product at *m/z* 149. **d** Mass spectrum of CCE reaction of two protonated acetone cations when the spray charged while the interface not charged. **e** Mass spectrum of CCE reaction of two protonated acetone cations when the spray and interface both charged. **f** Tandem mass spectrum of CCE product at *m/z* 105. RA denotes Relative Abundance. CCE: carbon chain elongation.

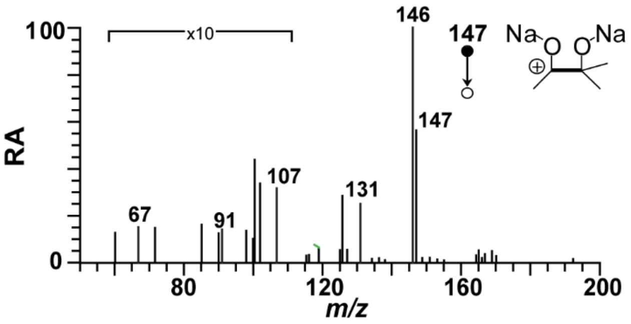

Supplementary Figure 10. Tandem mass spectrum of carbon chain elongation product at *m/z* 147 generated via the two-channel electrospray ionization online mass spectrometry. RA denotes Relative Abundance.

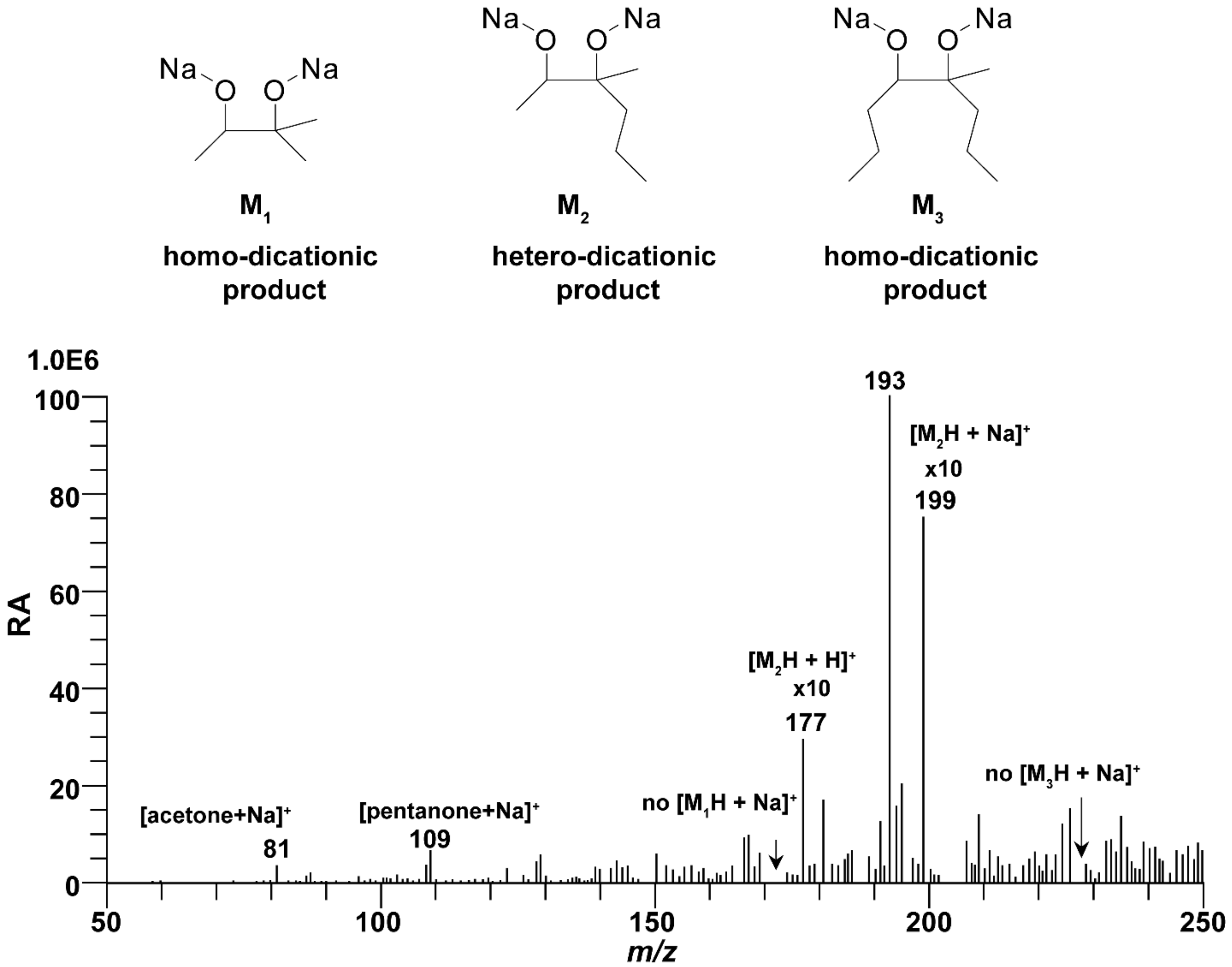

Supplementary Figure 11. Mass spectrum of collision reaction when sodiated acetone and sodiated pentanone were introduced to the spray and the interface, respectively. RA denotes Relative Abundance. M_1: homo-dicationic product. M_2: hetero-dicationic product. M_3: homo-dicationic product.

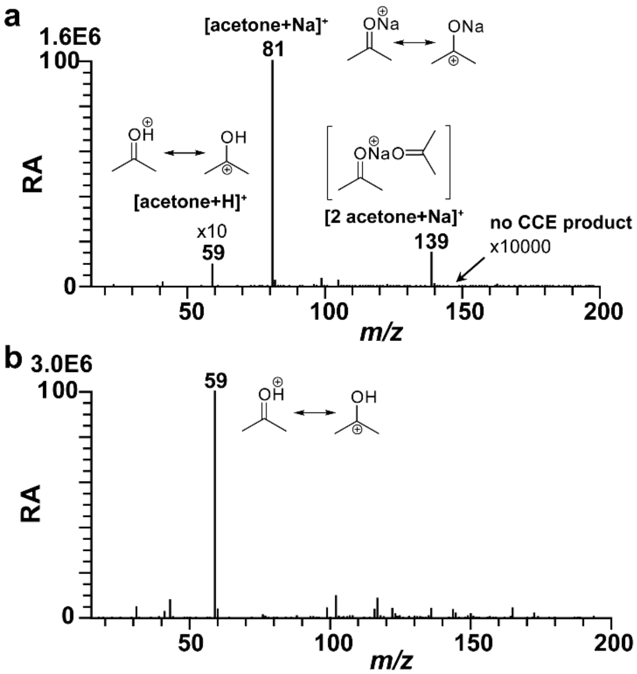

Supplementary Figure 12. Electrospray ionization mass spectrum of sample solution at the voltage of 6 kV. a Sodiated acetone aqueous solution. **b** Acetone aqueous solution. RA denotes Relative Abundance. CCE: carbon chain elongation.

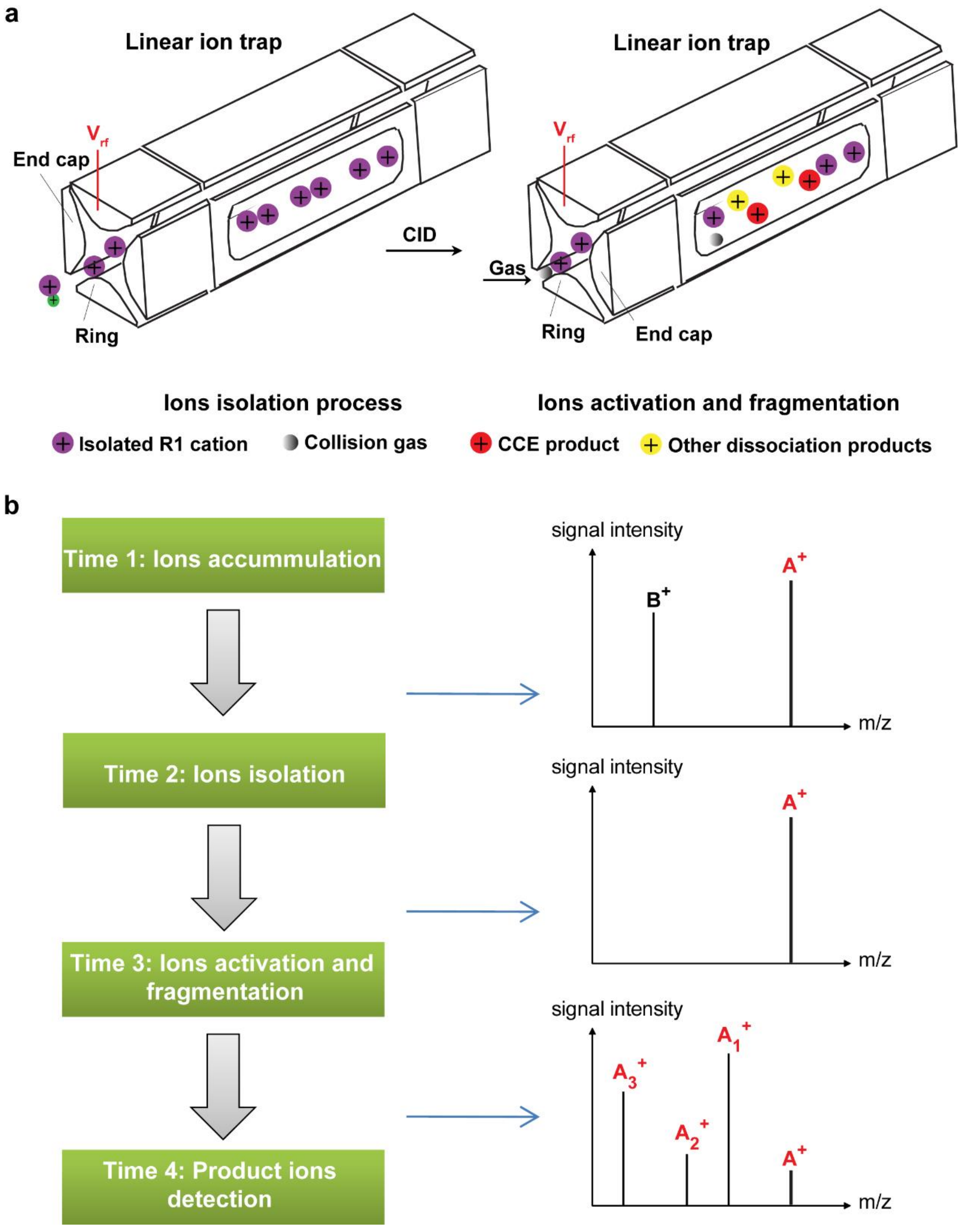

Supplementary Figure 13. Schematic picture of the process of carbon chain elongation (CCE) reaction occurring inside an ion trap. a The schematic illustration about the process of ion trap for gas phase CCE product formation. The figure is adapted with permission from ref. 9. **b** Process of multistage tandem mass spectrometry experiments in the ion trap. CID: collision-induced dissociation. R1: sodiated acetone.

Stable precursor ions can be transferred to the gas phase by electrospray ionization-based methods and then subjected to collision-induced dissociation (CID) to synthesize novel and reactive gas-phase ions. For convenience, further details and related discussions of the figure are listed below.

The schematic picture of the process of ion-trap experiments is shown in Supplementary Fig. 13a.The function of ion trap is mainly including ions accumulation and isolation, ions activation and fragmentation. The electrodes are ring electrode and end cap electrode, which are marked in the Supplementary Fig. 13a. In a typical CID process (mass analyzer), the electronics system in the instrument generates a broadband frequency spectrum with all resonating frequencies presented except for the frequency corresponding to the resonance of the target ion, isolating it for further analysis. After that, the energy of the target ion is increased by resonance excitation from the dipole field, and the resonating ions quickly take in energy from the dipolar field, collide with the helium background gas, and then dissociate to create a predictable and reproducible mass spectrum. The target ion will undergo thousands of collisions with neutral inert gas molecules, thereby converting its energy from kinetic to internal energy. Then the ions will dissociate into fragment ions or generate coupling products and were detected by mass spectrometry (MS). In addition, a neutral gas, such as acetone, is directly introduced into the ion trap through helium (Supplementary Fig. 13a), so that those neutral molecules were interacted with the ions isolated in the ion trap.

As shown in Supplementary Fig. 13b, the multistage tandem mass spectrometry experiments were performed in ion-trap instrument, where each MS step is separated in time. It allows the fragmentation of the product ions and thus produces next generation of product ions and so on. For example, in time 1, the normal electrospray ionization-mass spectrum (MS^1) is produced without fragmentation and most of ions can be obtained in this process. In time 2, one precursor ion such as sodiated acetone was isolated in the trap without collision energy, thus only sodiated acetone can be observed. In time 3, the sodiated acetone was activated by suitable collision energy and fragmented to produce the fragment ions, and the corresponding spectrum is called MS^2 spectrum. In time 4, one precursor is isolated from MS^2 spectrum and then fragmented to produce the product ions, which are scanned to record the MS^3 spectrum. Thus, the process of isolation and fragmentation can be repeated a number of times, resulting a series of MS^n spectra.

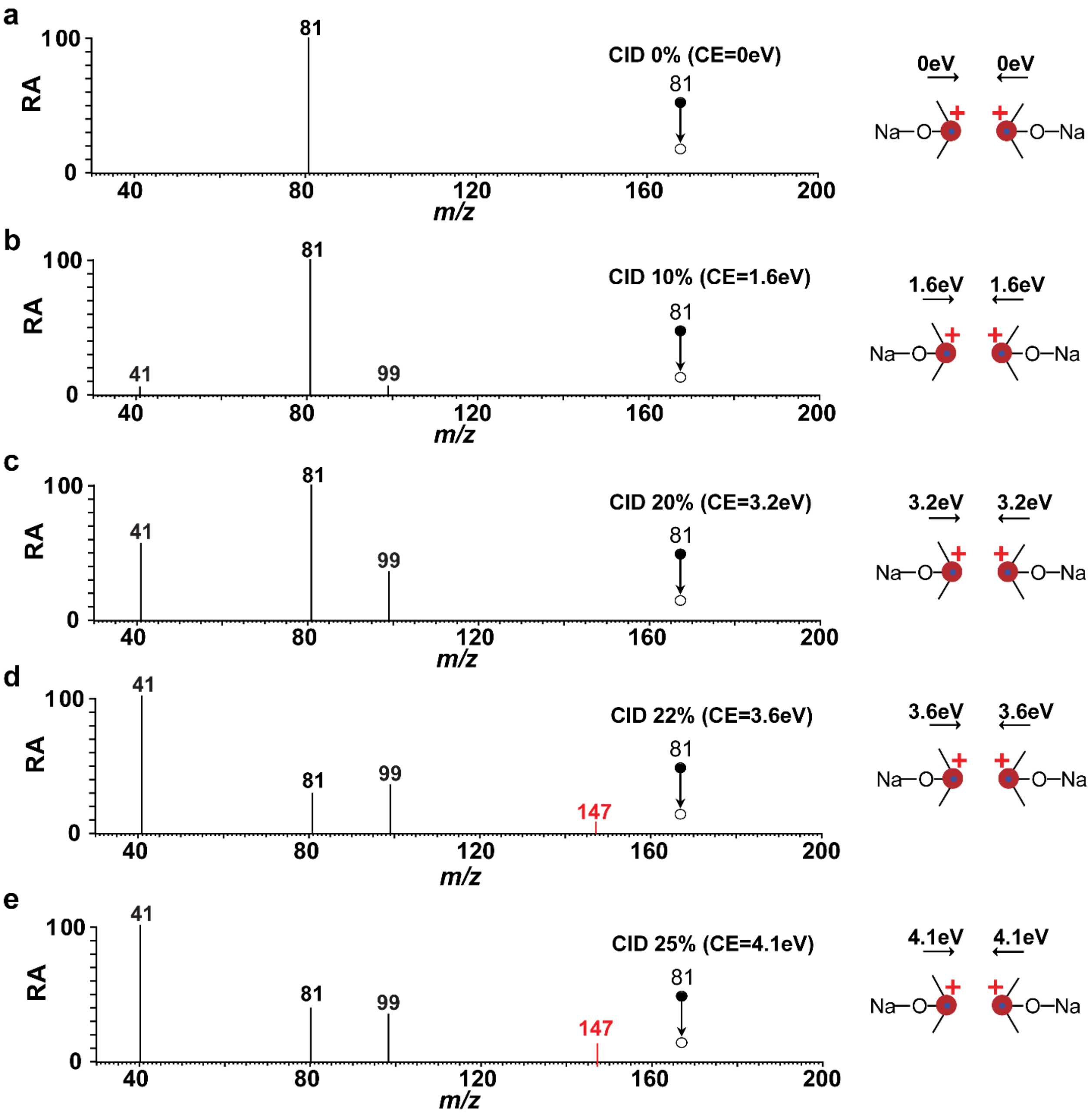

Supplementary Figure 14. Tandem mass spectra of sodiated acetone *m/z* 81 with different collision energies. a Collision energy with 0%. **b** Collision energy with 10%. **c** Collision energy with 20%. **d** Collision energy with 22%. **e** Collision energy with 25%. RA denotes Relative Abundance. CID: collision-induced dissociation. CE: collision energy.

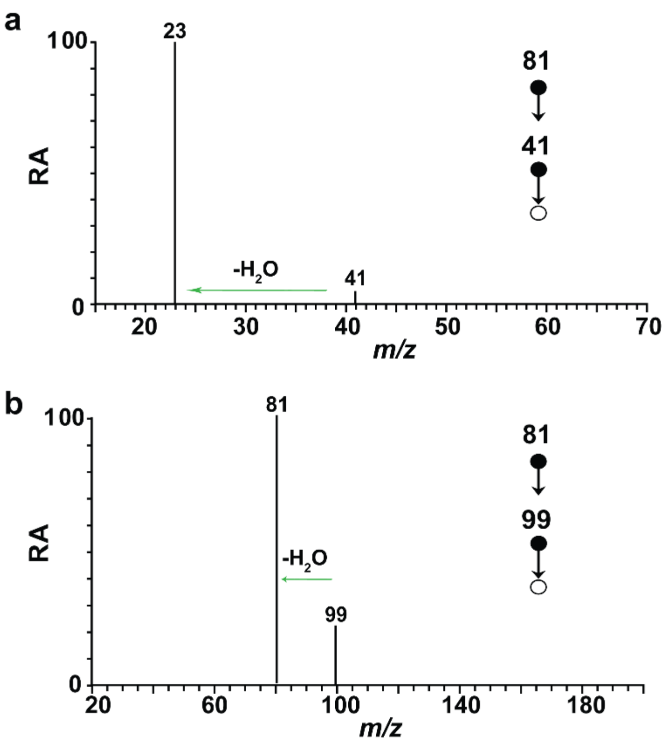

Supplementary Figure 15. Dissociation of sodiated acetone (R1, *m/z* 81). a Tandem mass spectrum of R1 (*m/z* 81 → *m/z* 41 →). **b** Tandem mass spectrum of R1 (*m/z* 81 → *m/z* 99 →). RA: Relative Abundance.

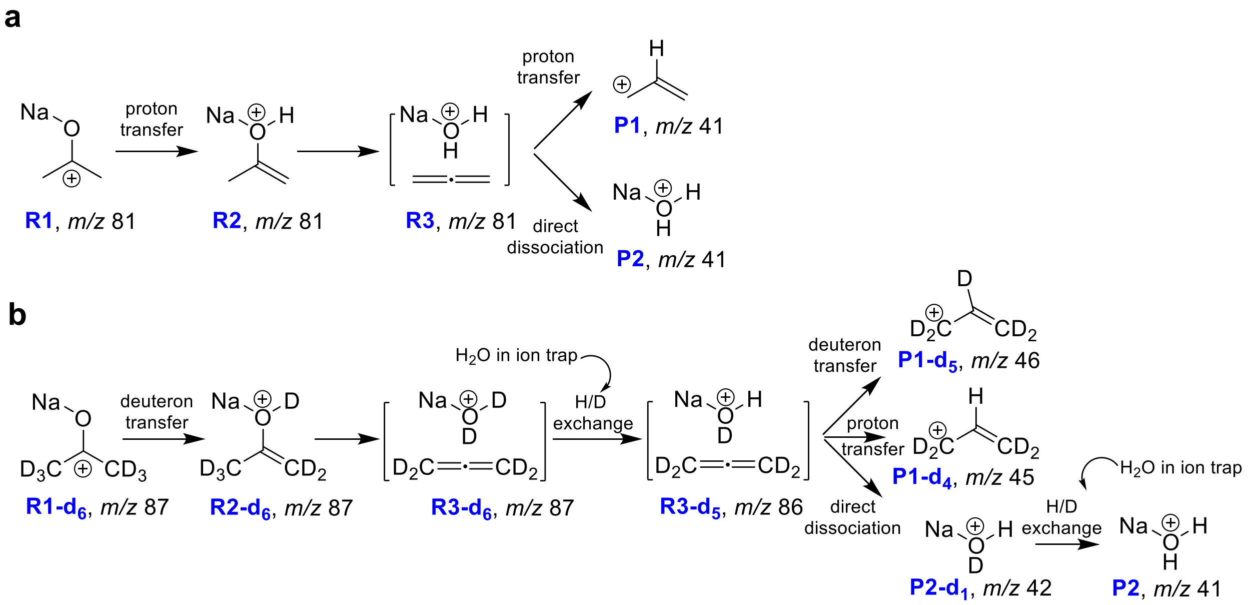

Supplementary Figure 16. Detailed process for the generation of P1 and P2 ions. a Dissociation pathway for generation of P1 and P2 (*m/z* 41) from R1 (*m/z* 81). **b** Dissociation pathways for generation of the corresponding P2 (*m/z* 41), P2-d_1 (*m/z* 42), P1-d_4 (*m/z* 45), and P1-d_5 (*m/z* 46) ions from sodiated acetone-d_6 (R1-d_6, *m/z* 87).

The peak at *m/z* 41 (Fig. 4b) designated the dissociation products, which were actually a mixture of the allyl cation (P1) and hydrated sodium cation (P2). As summarized in Supplementary Fig. 16, the P1 was produced via the proton transfer between the [hydrated sodium cation/alkene] ion-neutral complex (INC) R3, and the P2 was generated via direct dissociation of R3. The reaction of Supplementary Fig. 16a was supported by theoretical calculations (Supplementary Fig. 28a). Mass of P3 (*m/z* 99, Fig. 4b) shifted to *m/z* 105 (Fig. 4d) due to the mass shift of 6 units for R1-d_6 (*m/z* 87). The ionic fragment (R3-d_5) was recorded as the peak at *m/z* 86 (Fig. 4d), which was generated by H/D exchange between R3-d_6 and the water in the ion trap (Supplementary Fig. 16b). Similar to INC R3 (*m/z* 81), the corresponding INC R3-d_5 (*m/z* 86) underwent either deuteron transfer to form P1-d_5 ion (*m/z* 46), or proton transfer to form P1-d_4 ion (*m/z* 45), or direct dissociation to form P2-d_1 ion (*m/z* 42), or elimination of OH radical to form *m/z* 69 (Fig. 4d). The ionic P2-d_1 could further generate the P2 ions (*m/z* 41) by H/D exchange with the water in the ion trap (Fig. 4d). The intensity ratio of P1-d_4 (*m/z* 45) to P1-d_5 (*m/z* 46) was about 5:1, where the kinetic isotope effect of $k_H/k_D = 5$ was reported, confirming that *m/z* 41 is a mixture of the allyl cation (P1) and hydrated sodium cation (P2).

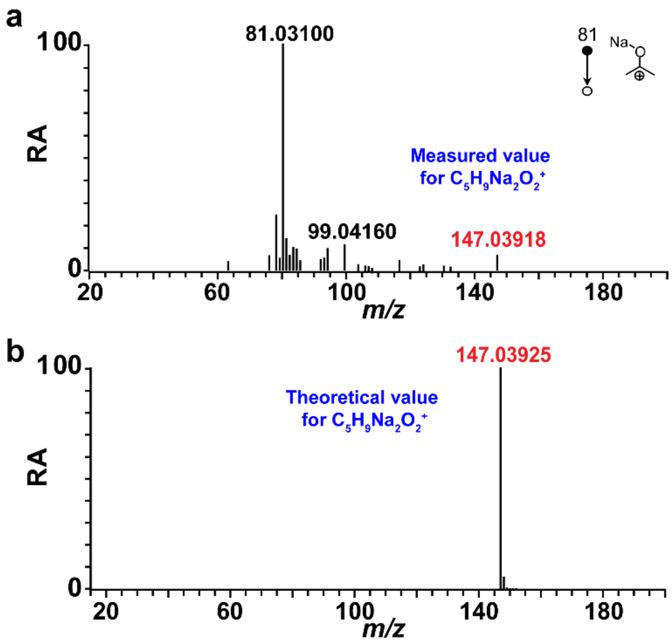

Supplementary Figure 17. The expected reaction product was detected at *m/z* 147, with the exact mass-to-charge ratio of *m/z* 147.03918 (Supplementary Fig. 17a), which ideally matched the theoretical value of $C_5H_9Na_2O_2^+$ (*m/z* 147.03925, Supplementary Fig. 17b) with an error of 0.5 ppm. The data show that the demethylation C–C coupling reaction was occurred inside an ion trap with high vacuum, suggesting that such reaction could be part of the intrinsic reactivity of the two sodiated acetone cations. RA: Relative Abundance.

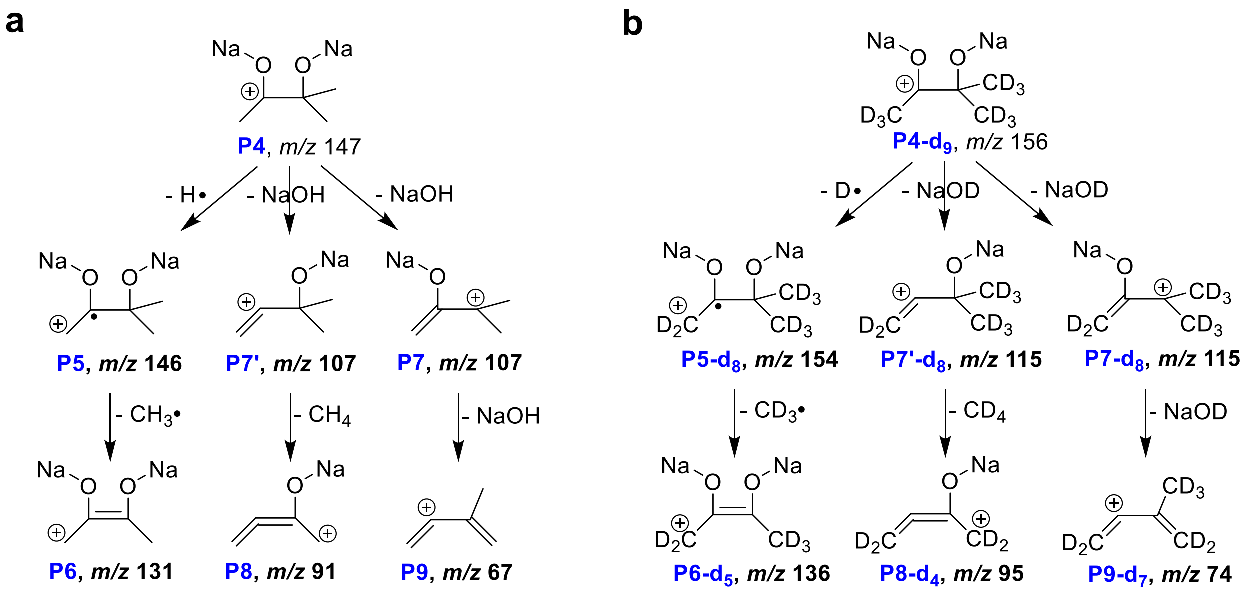

Supplementary Figure 18. Possible pathways for the fragmentation of P4, the product of the gas phase demethylation C–C coupling reaction. a Fragmentation of P4 ion (*m/z* 147). **b** Fragmentation of the P4-d_9 ion (*m/z* 156).

Supplementary Fig. 18a shows the plausible dissociation pathways of P4 (*m/z* 147) observed in the tandem mass spectrum of sodiated acetone (Fig. 4b), which were validated by detailed data recorded with multistage tandem mass spectrometry experiments (Fig. 4c). The fragment ions of P5 and P6 were formed by losing H radical and (H radical + CH_3 radical) from the precursor ion *m/z* 147, respectively. The fragment ions of *m/z* 107 in the tandem mass spectrum were generated by the elimination of NaOH from the precursor ion *m/z* 147, which might have two possible structures P7 and P7'. The product ions of P7 and P7' could undergo further dissociation to generate fragment ions at *m/z* 67 (P9) and *m/z* 91 (P8) with the elimination of NaOH and CH_4, respectively. The corresponding pathways for fragmentation of P4-d_9 (*m/z* 156) were elucidated in Supplementary Fig. 18b. The similar product ions *m/z* 154, *m/z* 136, *m/z* 115, *m/z* 95 and *m/z* 74 were formed by the elimination of D radical, (D radical + CD_3 radical), NaOD, (NaOD + CD_4), (NaOD + NaOD) from the precursor ion of *m/z* 156. Compared with Supplementary Fig. 18a, it showed that the dissociation pathways for fragmentation of P4 and P4-d_9 were reasonable. Thus, the experimental data supported that P4 (*m/z* 147) was sodium 2-methyl-butane-2,3-diolate)-3-ium.

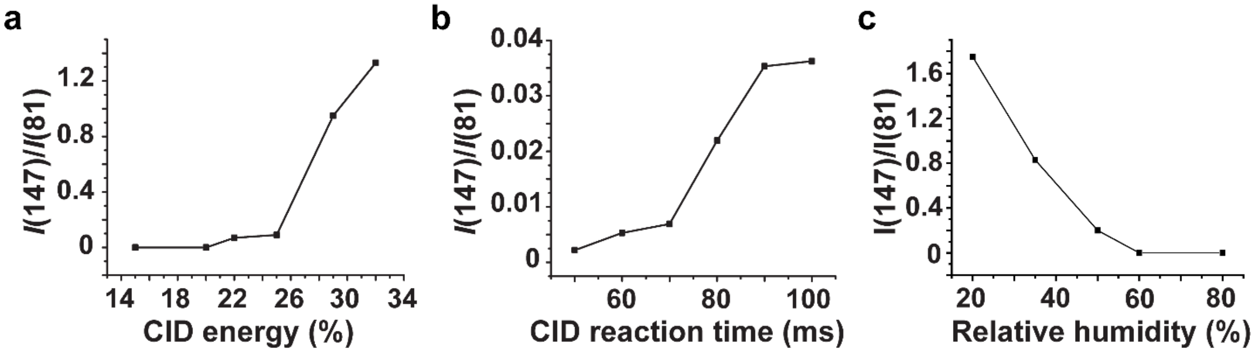

Supplementary Figure 19. The response of intensity ratio of P4 (*m/z* 147) and R1 (*m/z* 81) under different experimental conditions. a Ratio *I*(147) to *I*(81) *versus* different CID energy. **b** Ratio *I*(147)/*I*(81) *versus* different CID reaction time (collision energy=20%). **c** Ratio *I*(147)/*I*(81) *versus* different relative humidity of air. Note that the ion trap instrument was a commercial product. The conversion rate of the demethylation C–C coupling reaction could be further optimized using a totally customized reactor. CID: collision-induced dissociation. I: intensity.

It is noteworthy that the experimental conditions, such as collision energy (CE), collision-induced dissociation (CID) reaction time, and water vapour content inside the ion trap, might affect the formation of the *m/z* 147 ion. The intensity ratio of the *m/z* 147 to *m/z* 81 peaks increased as the CID energy changed from 16% to 35% (Supplementary Fig. 19a), indicating that the demethylation C–C coupling reaction between sodiated acetone cations occurred more easily with higher CID energy, probably because the sodiated acetone cations were better accelerated with higher collision energy. Similarly, the intensity ratio of the *m/z* 147 to *m/z* 81 peaks slowly increased in the CID reaction time range 50-70 ms, rapidly increased from 70 to 90 ms, and reached a plateau when the CID reaction time changed from 90 to 100 ms (Supplementary Fig. 19b). As the CID reaction time continued to increase, the intensity ratio of the *m/z* 147 to *m/z* 81 peaks may also increase slowly until it reaches the maximum at 200 ms. Note that the cations were distributed in a three-dimensional volume rather than being focused on the centre spot of the ion trap. When the reaction time gradually increased, the precursor ions underwent a number of collisions with neutral molecules, such as water, helium, and the presumably stationary singly charged species. However, only the collisions between the positive ions could lead to demethylation C–C coupling. Apparently, the neutral molecules in the ion trap prevent demethylation C–C coupling by reducing the repulsive Coulomb force between the cations. For example, as the relative humidity of the room air increased, the intensity ratio of the *m/z* 147 to *m/z* 81 peaks decreased rapidly (Supplementary Fig. 19c), because water molecules inside the ion trap preferably collided with the accelerated cations without notable energy barrier, particularly when the relative humidity of the room air was high (≥60%).

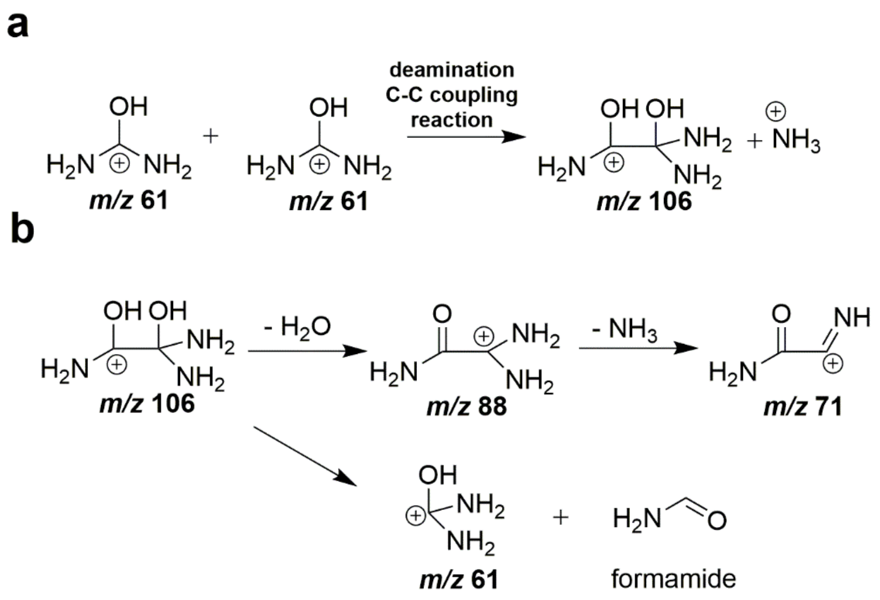

Supplementary Figure 20. The formation and fragmentation of *m/z* 106. a The formation process of *m/z* 106 from the tandem mass spectrum of protonated urea. **b** Possible pathways for fragmentation of *m/z* 106.

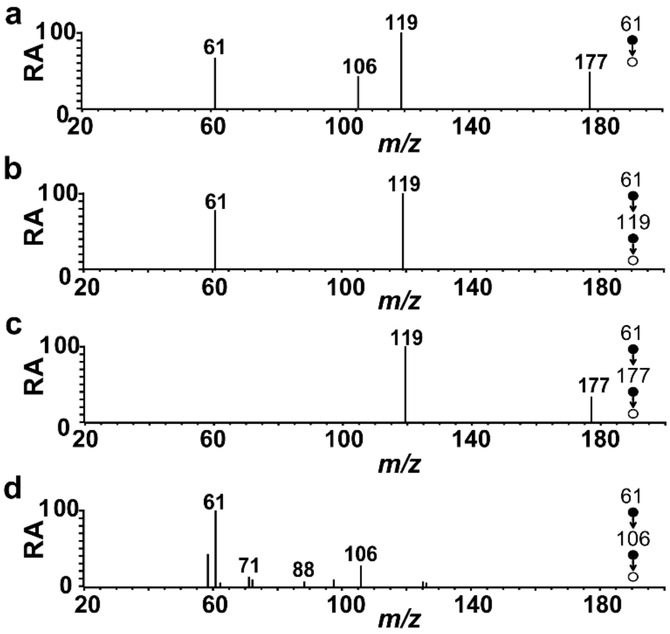

Supplementary Figure 21. The generation of ion-molecule adducts and deamination C–C coupling product via mass-selected protonated urea with acetone vapor in the gas phase (reaction time = 100 ms). **a** Tandem mass spectrum of protonated urea (*m/z* 61). The peak assignments: *m/z* 106, deamination C–C coupling product; *m/z* 119, [protonated urea + acetone]$^+$; *m/z* 177, [protonated urea + 2 acetone]$^+$. **b** Tandem mass spectrum of protonated urea (*m/z* 61 →*m/z* 119 →). **c** Tandem mass spectrum of protonated urea (*m/z* 61 →*m/z* 177 →). **d** Tandem mass spectrum of protonated urea (*m/z* 61 →*m/z* 106 →). Signal-to-noise ratio is significantly greater than 3. RA: Relative Abundance.

Extra experimental data confirmed that the repulsive Coulomb force between the two cations is necessary for demethylation C–C coupling. For instance, mass-selected protonated urea (*m/z* 61) interacted with acetone vapor (~10^{-3} Torr) in the ion trap to generate the ionic species of *m/z* 119 ([protonated urea + acetone]$^+$), *m/z* 177 ([protonated urea + 2acetone]$^+$) in the spectrum (Supplementary Fig. 21a). The tandem mass spectra data of *m/z* 119 (Supplementary Fig. 21b) and *m/z* 177 (Supplementary Fig. 21c) confirmed that all the species were proton-bound adducts of urea and acetone. No peak at *m/z* 104 (i.e., the ionic resultant) was observed, thus no expected demethylation C–C coupling reaction occurred between the protonated urea and neutral acetone. Interestingly, the peak at *m/z* 106 was produced by the reaction between two protonated urea cations in high (≥80%) yield, probably by the mechanism of deamination C–C coupling (Supplementary Fig. 20a). The deamination C–C coupling reaction was confirmed by tandem mass spectrum data of the ionic resultant (*m/z* 106, Supplementary Fig. 21d). The precursor ions of *m/z* 106 generated the characteristic fragments of *m/z* 88, *m/z* 71, and *m/z* 61 owing to the elimination of H_2O, (H_2O+NH_3), and formamide, respectively (Supplementary Fig. 20b).

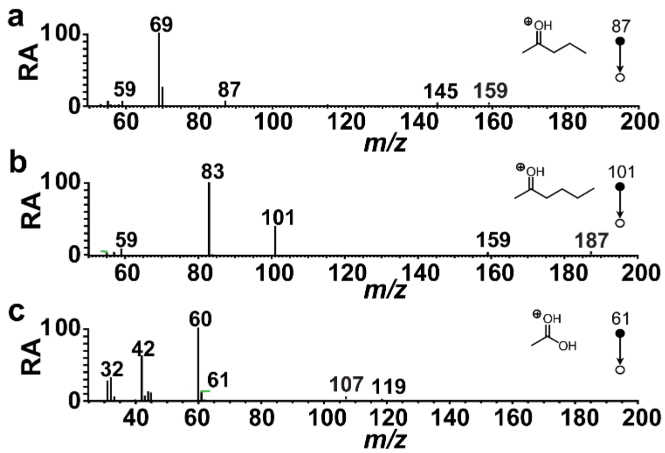

Supplementary Figure 22. The generation of ion–molecule adducts and demethylation C–C coupling product *via* mass-selected protonated compounds with acetone vapor in the gas phase (reaction time = 100 ms). a Tandem mass spectrum of protonated 2-pentanone (*m/z* 87). The peak assignments: *m/z* 159, demethylation C–C coupling product; *m/z* 145, [protonated 2-pentanone + acetone]$^+$; *m/z* 69, elimination of H_2O from *m/z* 87. **b** Tandem mass spectrum of protonated 2-hexanone (*m/z* 101). The peak assignments: *m/z* 187, demethylation C–C coupling product; *m/z* 159, [protonated 2-hexanone + acetone]$^+$; *m/z* 83, elimination of H_2O from *m/z* 101. **c** Tandem mass spectrum of protonated acetic acid (*m/z* 61). The peak assignments: *m/z* 107, demethylation C–C coupling product; *m/z* 119, [protonated acetic acid + acetone]$^+$; *m/z* 42, elimination of H_2O from *m/z* 60. RA: Relative Abundance.

Supplementary Fig. 22 shows the generation of ion-molecule adducts and demethylation C–C coupling products *via* ion-molecule reaction of mass-selected protonated compounds such as protonated 2-pentanone, protonated 2-hexanone, and protonated acetic acid with acetone vapor (reaction time = 100 ms). Tandem mass spectrum of protonated 2-pentanone (*m/z* 87) generated product ions at *m/z* 69, *m/z* 70, *m/z* 145, and *m/z* 159. In which product ions at *m/z* 69, *m/z* 70 and *m/z* 145 were formed by elimination of H_2O, elimination of OH radical and addition of acetone vapor from protonated 2-pentanone, respectively. Product ion *m/z* 159 was formed by demethylation C–C coupling reaction between two protonated 2-pentanone (Supplementary Fig. 22a). Similar ion-molecule adducts ([protonated 2-hexanone + acetone]$^+$, *m/z* 159; [protonated acetic acid + acetone]$^+$, *m/z* 119) and demethylation C–C coupling products (*m/z* 187 and *m/z* 107) were obtained *via* ion-molecule reaction of mass-selected protonated 2-hexanone (*m/z* 101) and protonated acetic acid (*m/z* 61) with neutral acetone (Supplementary Figs. 22b,c). Thus, these findings further confirmed that the repulsive Coulomb force is necessary for demethylation/deamination C–C coupling reactions. In general, mass-selected protonated compounds interacted with acetone vapor (~10^{-3} Torr) in the ion trap to generate the ionic species of [protonated compounds + acetone]$^+$, and the demethylation C–C coupling products between two protonated compounds in the spectrum, while no expected demethylation C–C coupling reaction occurred between the protonated compounds and neutral acetone. Therefore, it further confirmed that the demethylation C–C coupling reaction was occurred between two positive cations, not between positive cations and neutral compounds.

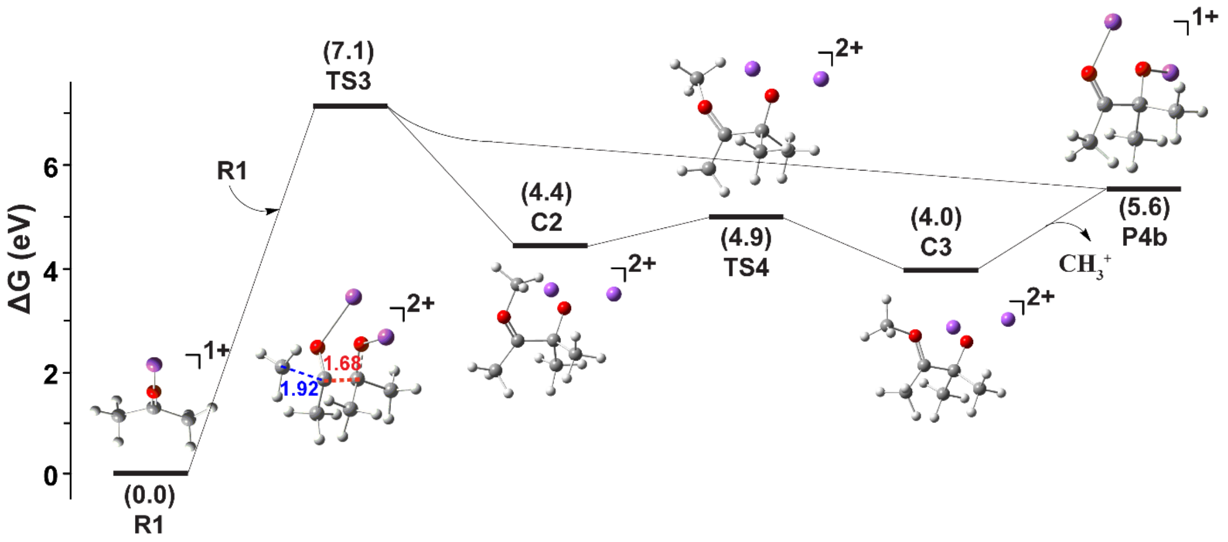

Supplementary Figure 23. The stationary points and energies on the minimum energy path for additional pathway that support the demethylation C–C coupling reaction process. Relative energy levels to generate P4b ion via TS3 by the demethylation C–C coupling reaction. Purple Na, red O, gray C, and white H. R1: *m/z* 81, P4b: *m/z* 147.

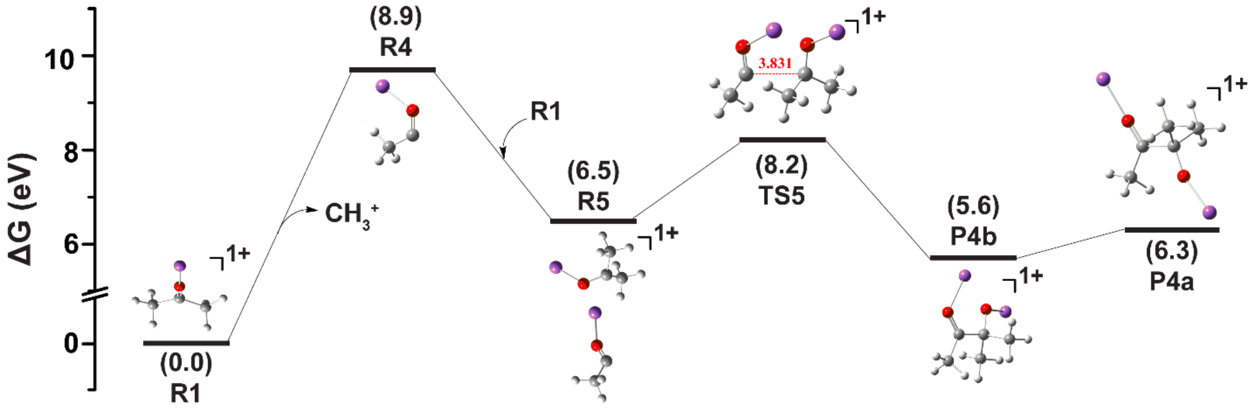

Supplementary Figure 24. Relative energy levels for route 2 to generate P4a by the ion-molecule reaction. The unit of the bond distances is angstroms (Å). Purple Na, red O, gray C, and white H. R1: *m/z* 81, R4: CH_3NaOC:, P4a, P4b: *m/z* 147. For convenience, further details and related discussions of the figure are listed below.

As shown in Supplementary Fig. 24, a carbene intermediate R4 and a methyl cation must first be generated through the dissociation of the sodiated acetone R1 before the reaction could proceed, with an endergonic process of 8.9 eV. After that, the carbene intermediate R4 interacts with another R1 to form the ion-neutral complex R5, in which the ion and neutral species were temporarily trapped by electrostatic interactions. The intermediate R5 may undergo an ion-molecule reaction to generate P4b via TS5 with an energy barrier of 1.7 eV. Compared with Route 1, the energy needed to yield P4a and CH_3^+ via the two-step mechanism in Route 2 (~8.9 eV) is at least 1.7 eV higher than that via the direct mechanism in Route 1 (7.2 eV), indicating that the reaction via the direct mechanism is a kinetically more favourable process.

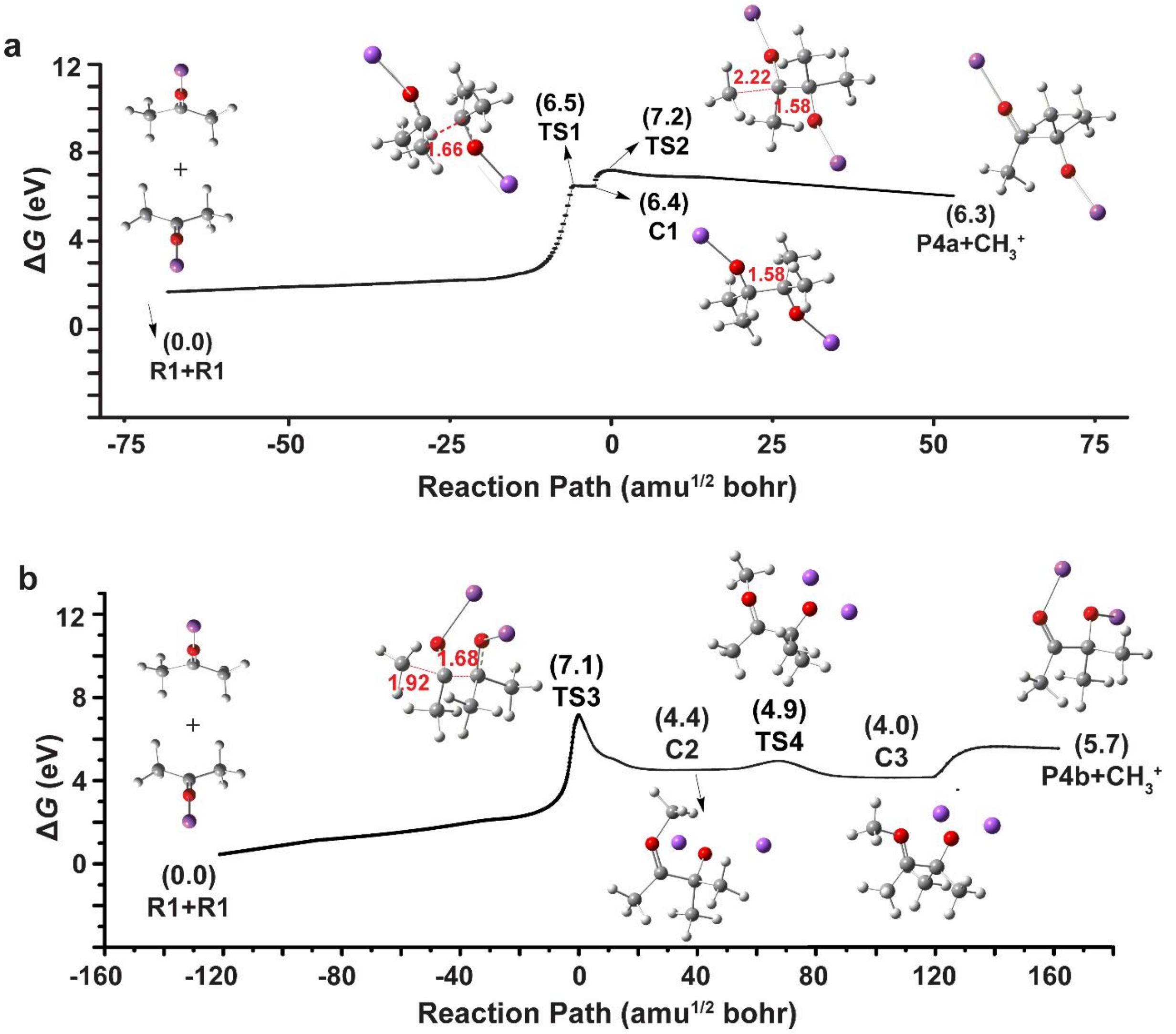

Supplementary Figure 25. Intrinsic reaction coordinate (IRC) calculations of Route 1a via TS1, TS2 and Route 1b via TS3, C1, TS4, C2 for the formation of product ion P4 (two configurations including P4a, P4b) *m/z* 147 at the B3LYP-gcp-D3/6-31+G(d) level. a IRC calculations of TS1 and TS2 for the formation of product ion P4a at *m/z* 147. **b** IRC calculations of TS3 and TS4 for the formation of product ion P4b at *m/z* 147.

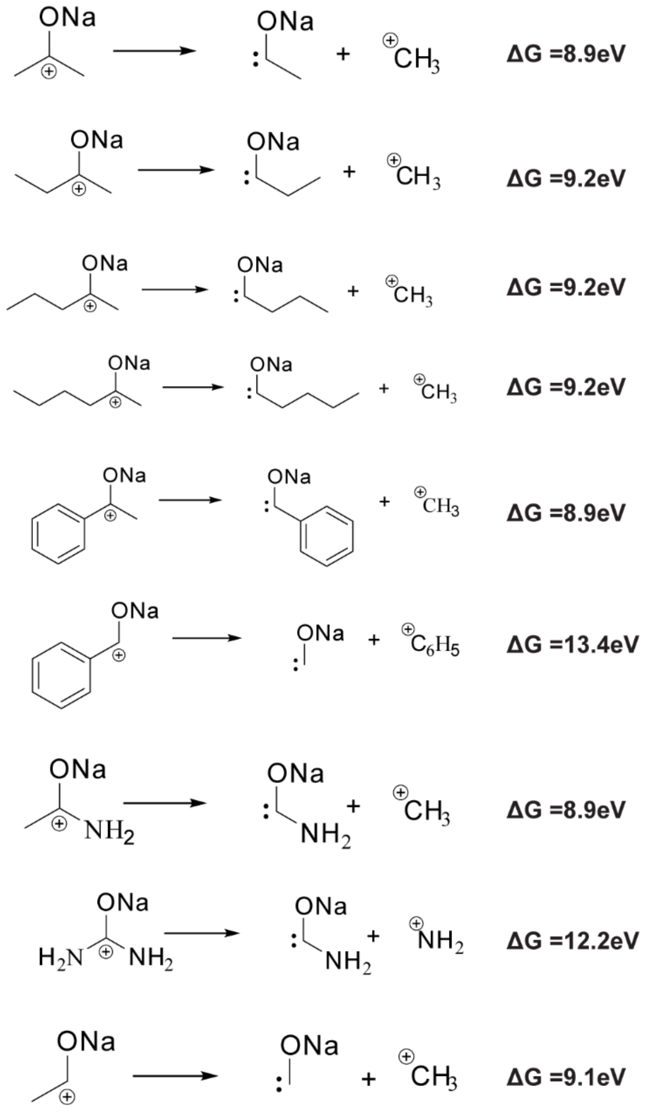

Supplementary Figure 26. The dissociation energies for different compounds.

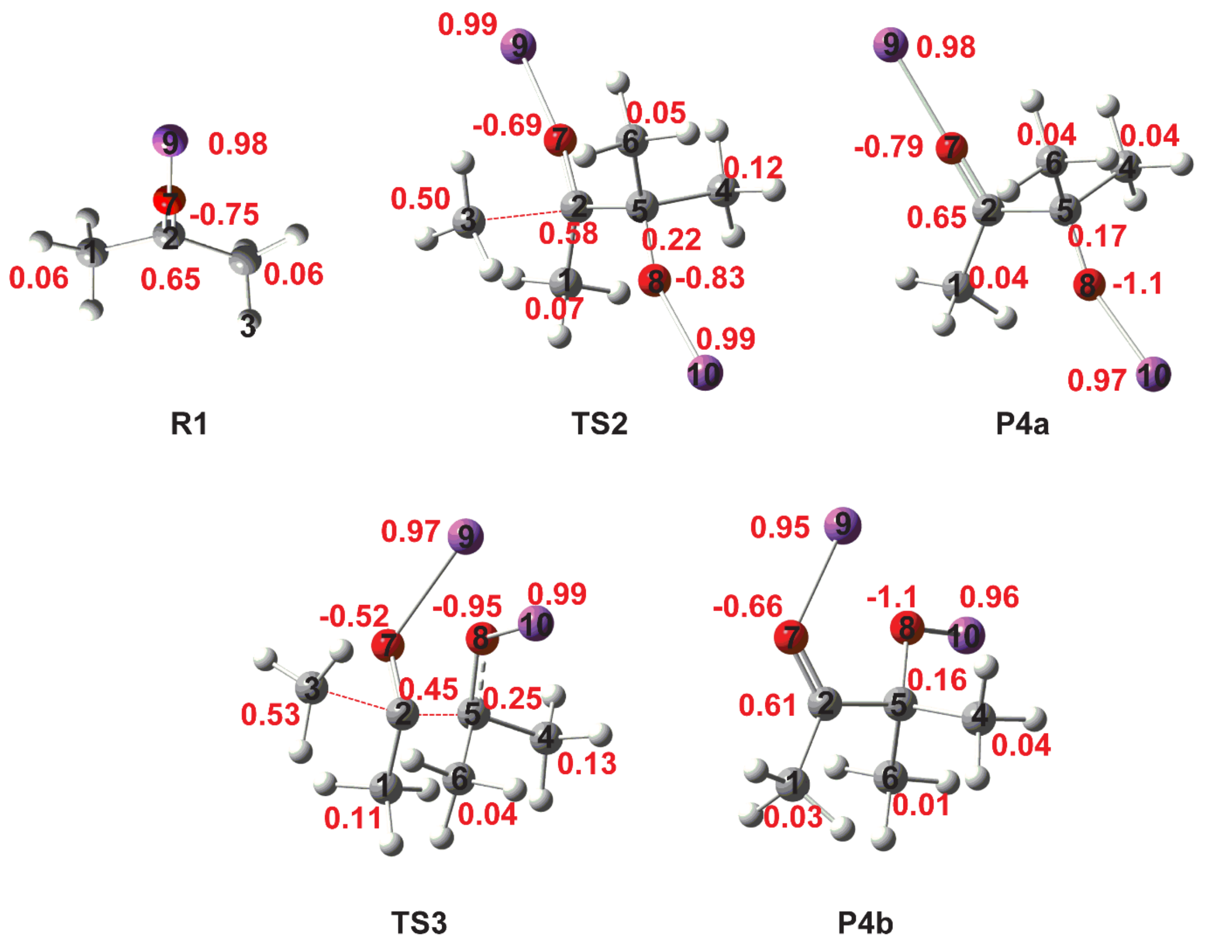

Supplementary Figure 27. Natural population analysis of R1, TS2, P4a, TS3 and P4b. It shows the charge distribution of some key structures involving in CCE reaction calculated by using the natural bond orbital method. It was found that C2 atom (～0.65 |e|) and Na9 atom (～0.98 |e|) in R1 and P4a possess the mainly positive charge due to the delocalization of charge. Obviously, when two R1 cations were closed to each other and reached the cross-section of them through TS2, the positive charge on C2 atom and C5 atom decreases while positive charge on C3 atom and C4 atom increases, indicating the charge transfer from carbonyl carbon to methyl carbon, presumably due to the large repulsive Coulomb force between two cations (as shown in Fig. 7a).

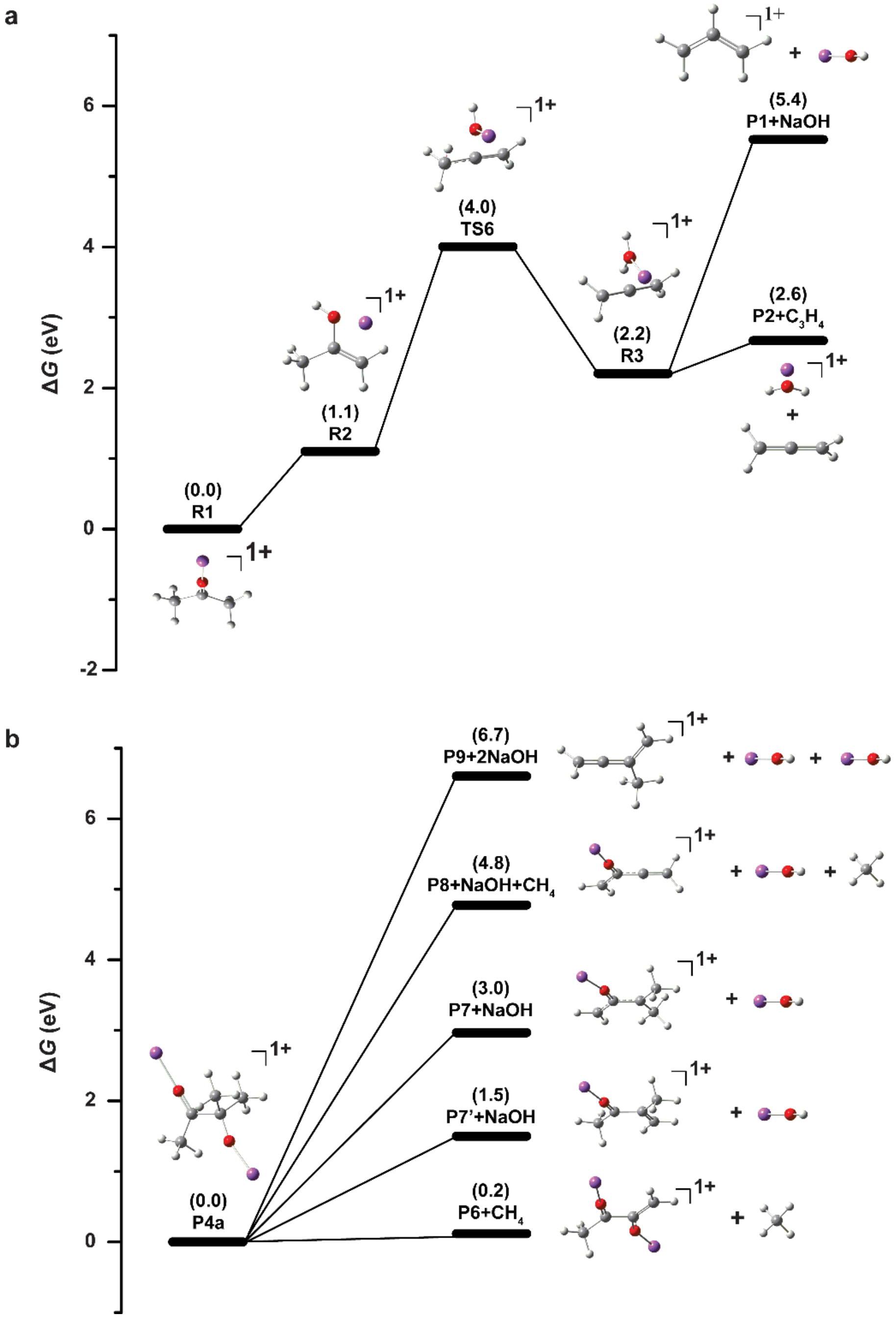

Supplementary Figure 28. Relative energy levels of the product ions. a Relative energies of product ions involved in dissociation of R1 (*m/z* 81). **b** Relative energies of the product ions involved in dissociation of P4 (*m/z* 147). The unit of the bond distance is angstroms (Å). Purple Na, red O, gray C, and white H. R1: *m/z* 81, R2: *m/z* 81, R3: *m/z* 81, P1: *m/z* 41. P2: *m/z* 41. P4a: *m/z* 147, P6: *m/z* 131, P7: *m/z* 107, P8: *m/z* 91, P7': *m/z* 107, P9: *m/z* 67.

Supplementary Fig. 28a shows the relative energy levels required by P1 (*m/z* 41) and P2 (*m/z* 41) obtained by dissociation of sodiated acetone (R1, *m/z* 81). R1 is the global minimum on the

calculated potential energy curve. The stability of R1 is a consequence of the resonance in the planar sodium carbonyl structure, which disperses the positive charge to the carbonyl carbon and thus activates the hydrogen atom in the methyl group. The activated proton in R1 can easily be transferred from the methyl carbon to the oxygen by keto–enol tautomerism, leading to formation of R2 with energy of 1.1 eV. In R2, the positively charged oxygen atom induces the cleavage of the C–O bond and migration of a proton from the methyl carbon to oxygen *via* transition state TS6, leading to formation of INC R3 [hydrated sodium cation/alkene]. The energy barrier of TS6 is 2.9 eV higher than the energy of intermediate R2, which suggests that formation of R3 can be achieved in the ion trap. The sum of the free energies of P1 and NaOH is 2.8 eV more than that of P2 and alkene. However, the minimum internal excess energy of R3 is 1.8 eV, which could induce R3 to undergo further activation to form thermodynamically unfavorable P1. Thus, the product ions at *m/z* 41 are P2 and P1, which is in good agreement with the collision-induced dissociation mass spectrometry experimental results (Fig. 4b). As shown in Supplementary Fig. 28b, the energies required to dissociate P4 (*m/z* 147) were ordered as: 0.2 eV (P6 + CH_4) < 1.5 eV (P7' + NaOH) < 3.0 eV (P7 + NaOH) < 4.8 eV (P8 + NaOH + CH_4) < 6.7 eV (P9 + 2NaOH). The energy order was in agreement with the experimental measurements (Fig. 4c).

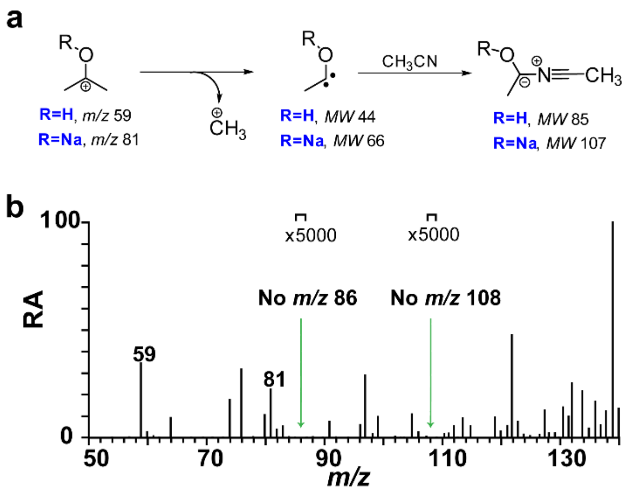

Supplementary Figure 29. Data supporting no capture of carbene intermediate by CH_3CN, demonstrating that the Route 2 was unlikely to generate the CCE product obtained by the setup shown in Fig. 2a. a Schematic illustration on the process of the capture of carbene intermediate by CH_3CN. **b** MS spectrum of CCE reaction of protonated/sodiated acetone by adding CH_3CN to capture carbene intermediate (CH_3HOC:, MW 44; CH_3NaOC:, MW 66) using the experimental setup shown in Fig. 2a. No targeted ions at *m/z* 86 (CH_3CN capture CH_3HOC:) or *m/z* 108 (CH_3CN capture CH_3NaOC:) are detected. For convenience, further details and related discussions of the figure are listed below. RA: Relative Abundance. CCE: carbon chain elongation.

As indicated in Supplementary Fig. 29a, a nitrile ylide intermediate can be formed when adding the CH_3CN solvent to carbene intermediate. In our study, carbene intermediate will be generated if the formation of *m/z* 147 is through the ion/molecule reaction (Route 2 in Fig. 5b). To further experimentally determine the possible reaction mechanism for the formation of *m/z* 147, an experiment was performed for the capture of carbene intermediates using the experimental setup shown in Fig. 2a, with CH_3CN as the capturing reagent. Supplementary Fig. 29b shows the capture of carbene intermediate by CH_3CN using protonated/sodiated acetone as the reactants. If carbene intermediate is generated, then the products nitrile ylide should be obtained (Supplementary Fig. 29a). It can be found that no targeted nitrile ylide ions at *m/z* 86 (CH_3CN capture CH_3HOC:) or *m/z* 108 (CH_3CN capture CH_3NaOC:) are detected (Supplementary Fig. 29b). Therefore, the formation of *m/z* 147 is likely through the carbon chain elongation reaction by elimination of a methyl cation with a direct mechanism, which is in accordance with the theoretical calculation results.

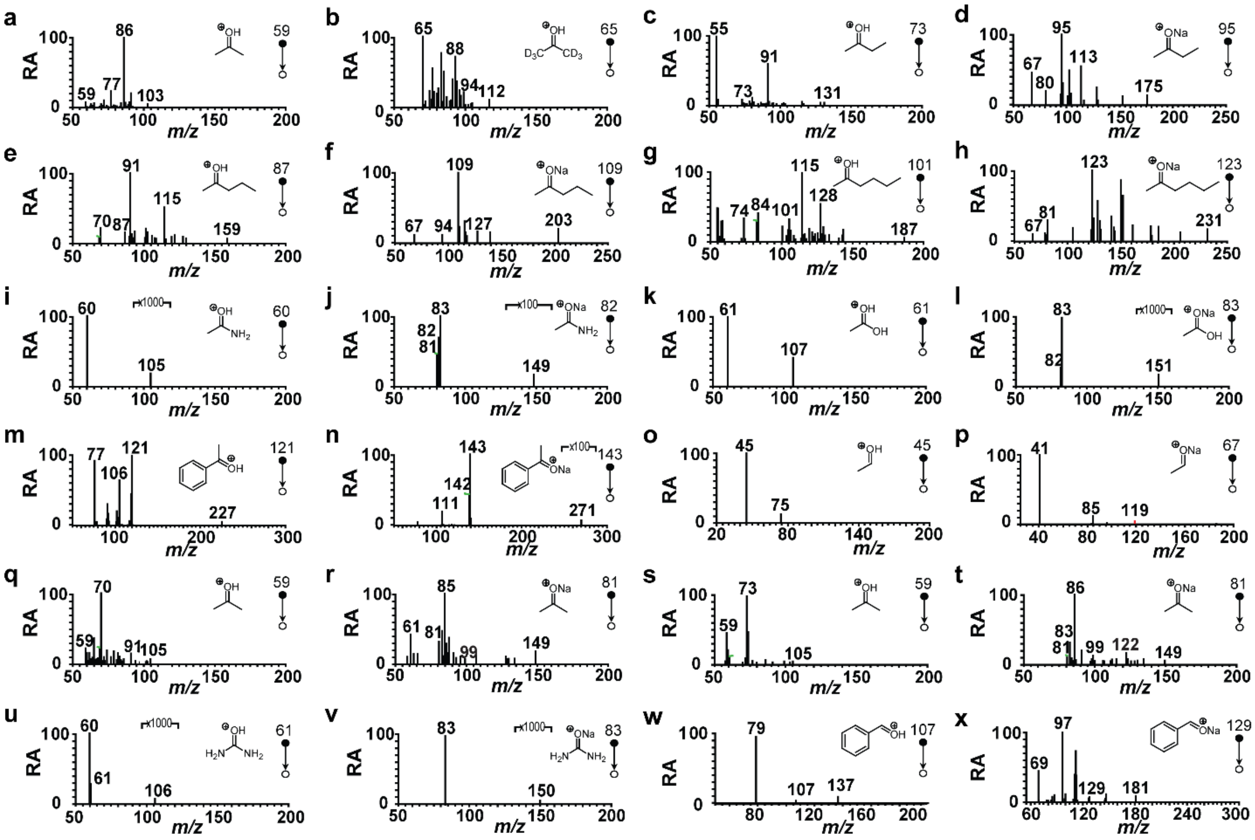

Supplementary Figure 30. Tandem mass spectra of the sodiated/protonated species of different compounds. **a** Protonated acetone (*m/z* 59). **b** Protonated acetone-d$_6$ (*m/z* 65). **c** Protonated butanone (*m/z* 73). **d** Sodiated butanone (*m/z* 95). **e** Protonated 2-pentanone (*m/z* 87). **f** Sodiated 2-pentanone (*m/z* 109). **g** Protonated 2-hexanone (*m/z* 101). **h** Sodiated 2-hexanone (*m/z* 123). **i** Protonated acetamide (*m/z* 60). **j** Sodiated acetamide (*m/z* 82). **k** Protonated acetic acid (*m/z* 61). **l** Sodiated acetic acid (*m/z* 83). **m** Protonated acetophenone (*m/z* 121). **n** Sodiated acetophenone (*m/z* 143). **o** Protonated acetaldehyde (*m/z* 45). **p** Sodiated acetaldehyde (*m/z* 67). **q** Protonated acetone (*m/z* 59), urea was added into the solution. **r** Sodiated acetone (*m/z* 81), urea was added into the solution. **s** Protonated acetone (*m/z* 59), acetic acid was added into the solution. **t** Sodiated acetone (*m/z* 81), acetic acid was added into the solution. **u** Protonated urea (*m/z* 61). **v** Sodiated urea (*m/z* 83). **w** Protonated benzaldehyde (*m/z* 107). **x** Sodiated benzaldehyde (*m/z* 129). For convenience, further details and related discussions of the figure are listed below. RA: Relative Abundance.

Supplementary Fig. 30 shows the tandem mass spectra of the sodiated/protonated species of different compounds. Upon collision-induced dissociation, the tandem mass spectrum of protonated acetone at *m/z* 59 generated product ion at *m/z* 103, which was the product of gas-phase demethylation C–C coupling reaction between two protonated acetone ions at *m/z* 59. In general, the products of gas-phase demethylation C–C coupling reaction were observed either in the dissociation of sodiated species or in the dissociation of protonated species of different compounds

such as butanone, 2-pentanone, 2-hexanone, acetamide, acetic acid, acetophenone, acetaldehyde, and ketones reacted with other molecules (e.g., urea, acetic acid). The products of gas-phase deamination C–C coupling and gas-phase dephenylation C–C coupling reactions were both observed in the dissociation of protonated/sodiated urea and protonated/sodiated benzaldehyde, respectively. Therefore, it can be concluded that the demethylation/deamination/dephenylation C–C coupling reactions catalyzed by the strong repulsive Coulomb force between two positively charged organic species would be of wide universality for various compounds. As demonstrated using various compounds with different groups, the strategy favored rapid molecule construction with a wide substrate scope and environment-friendly conditions.

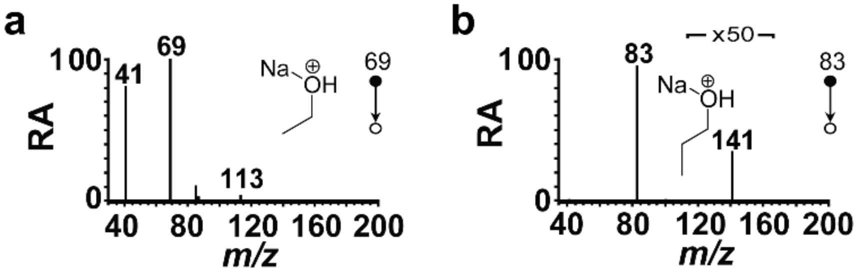

Supplementary Figure 31. Tandem mass spectra of the sodiated species of ethanol and propanol. a Sodiated ethanol (*m/z* 69). **b** Sodiated propanol (*m/z* 83). RA: Relative Abundance.

Supplementary Table 1 | Targeted carbon chain elongation (CCE) reaction occurred only between two positively charged organic species in the liquid phase under ambient conditions.

Reactant a		Reactant b		C–C coupling product ions	Leaving group	Final stable products of CCE	Yields
Neutral form	Charged form	Neutral form	Charged form				
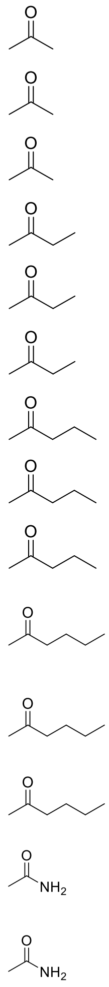	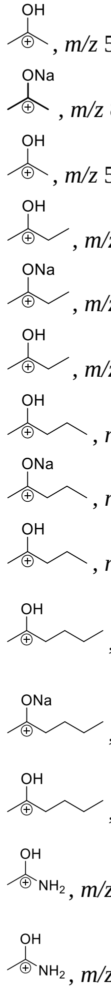, *m/z* 59	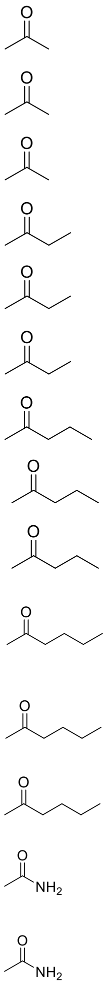	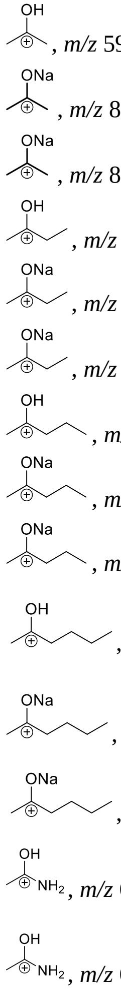, *m/z* 59	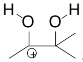, *m/z* 103	CH_3^+		
	, *m/z* 81		, *m/z* 81	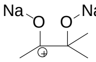, *m/z* 147	CH_3^+	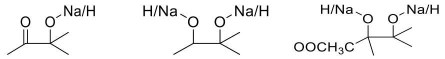	14%
	, *m/z* 59		, *m/z* 81	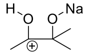, *m/z* 125	CH_3^+		
	, *m/z* 73		, *m/z* 73	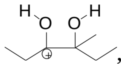, *m/z* 131	CH_3^+		
	, *m/z* 95		, *m/z* 95	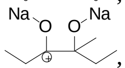, *m/z* 175	CH_3^+	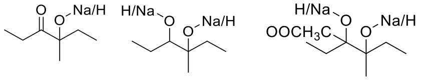	45%
	, *m/z* 73		, *m/z* 95	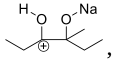, *m/z* 163	CH_3^+		
	, *m/z* 87		, *m/z* 87	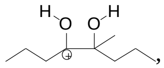, *m/z* 159	CH_3^+		
	, *m/z* 109		, *m/z* 109	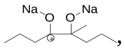, *m/z* 203	CH_3^+	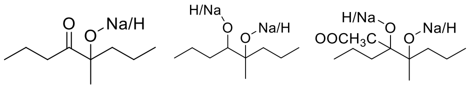	23.6%
	, *m/z* 87		, *m/z* 109	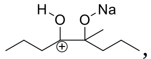, *m/z* 181	CH_3^+		
	, *m/z* 101		, *m/z* 101	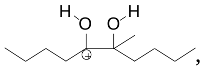, *m/z* 187	CH_3^+		
	, *m/z* 123		, *m/z* 123	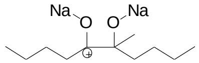, *m/z* 231	CH_3^+	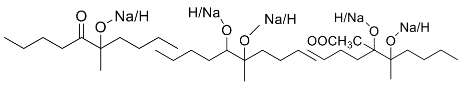	10.8%
	, *m/z* 101		, *m/z* 123	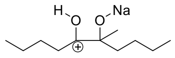, *m/z* 209	CH_3^+		
	, *m/z* 60		, *m/z* 60	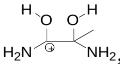, *m/z* 105	CH_3^+	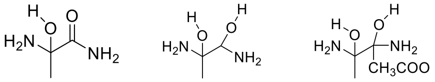	0.8%
	, *m/z* 60		, *m/z* 60	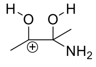, *m/z* 104	NH_2^+	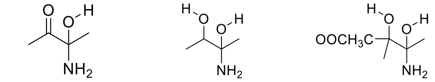	

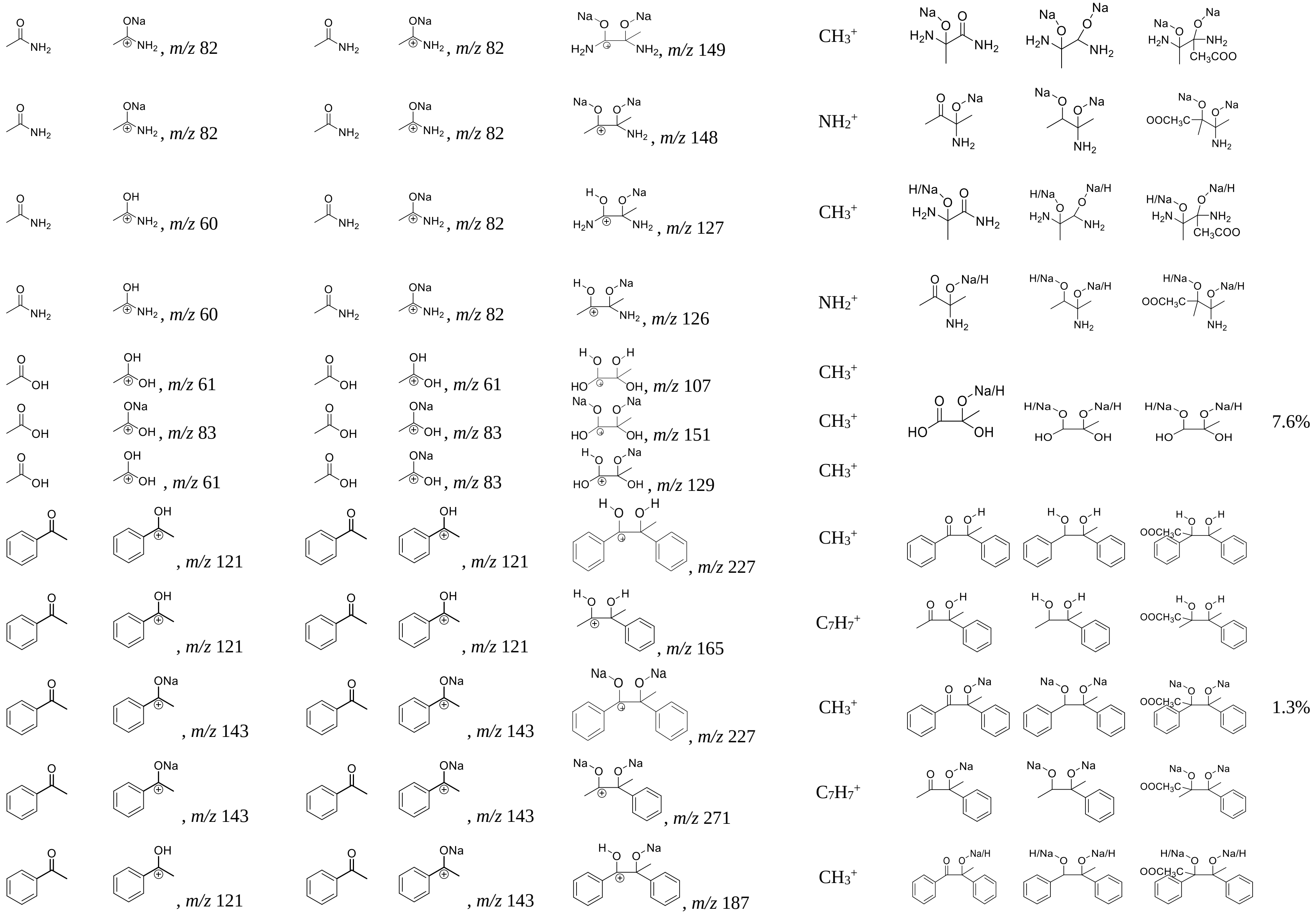

, m/z 82
, m/z 82
, m/z 149
CH3+
, m/z 82
, m/z 82
, m/z 148
NH2+
, m/z 60
, m/z 82
, m/z 127
CH3+
, m/z 60
, m/z 82
, m/z 126
NH2+
, m/z 61
, m/z 61
, m/z 107
CH3+
, m/z 83
, m/z 83
, m/z 151
CH3+
7.6%
, m/z 61
, m/z 83
, m/z 129
CH3+
, m/z 121
, m/z 121
, m/z 227
CH3+
, m/z 121
, m/z 121
, m/z 165
C7H7+
, m/z 143
, m/z 143
, m/z 227
CH3+
1.3%
, m/z 143
, m/z 143
, m/z 271
C7H7+
, m/z 121
, m/z 143
, m/z 187
CH3+

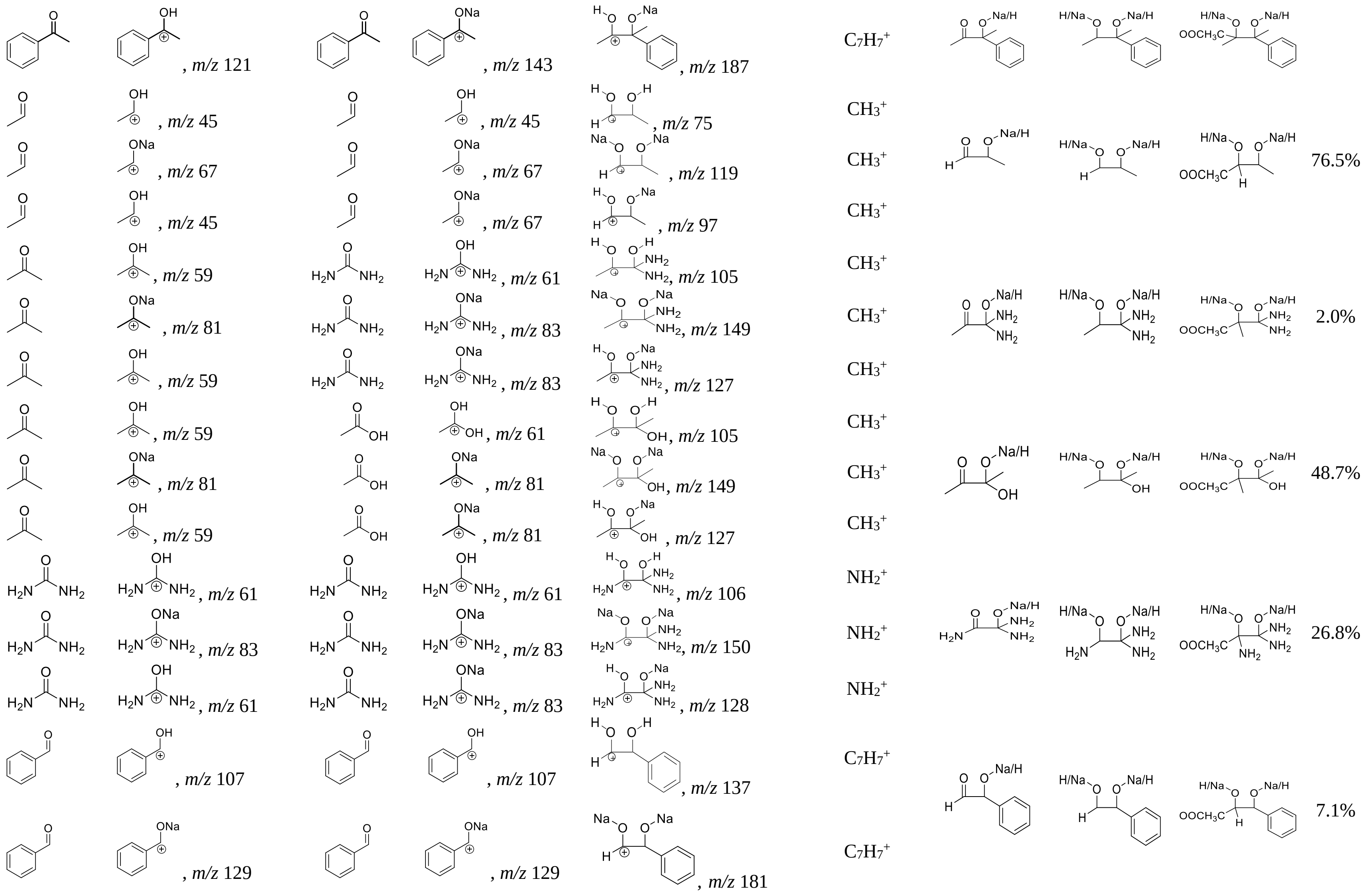
, m/z 121
, m/z 143
, m/z 187
C7H7+
, m/z 45
, m/z 45
, m/z 75
CH3+
, m/z 67
, m/z 67
, m/z 119
CH3+
76.5%
, m/z 45
, m/z 67
, m/z 97
CH3+
, m/z 59
, m/z 61
, m/z 105
CH3+
, m/z 81
, m/z 83
, m/z 149
CH3+
2.0%
, m/z 59
, m/z 83
, m/z 127
CH3+
, m/z 59
, m/z 61
, m/z 105
CH3+
, m/z 81
, m/z 81
, m/z 149
CH3+
48.7%
, m/z 59
, m/z 81
, m/z 127
CH3+
, m/z 61
, m/z 61
, m/z 106
NH2+
, m/z 83
, m/z 83
, m/z 150
NH2+
26.8%
, m/z 61
, m/z 83
, m/z 128
NH2+
, m/z 107
, m/z 107
, m/z 137
C7H7+
7.1%
, m/z 129
, m/z 129
, m/z 181
C7H7+

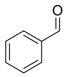	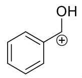 , *m/z* 107	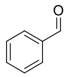	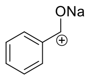 , *m/z* 129	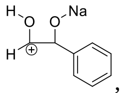 , *m/z* 159	$C_7H_7^+$

Supplementary Table 2. No targeted carbon chain elongation (CCE) reaction occurred between positively charged organic species and neutral species in the liquid phase under ambient conditions.

Reactant a		Reactant b	C–C coupling product ions	Leaving group	Final stable product of CCE	Yields
Neutral form	Charged form	Neutral form				
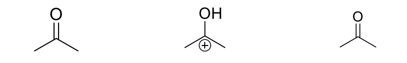			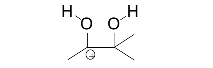	CH_3^+	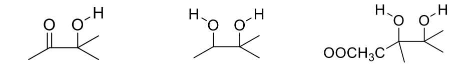	N/A
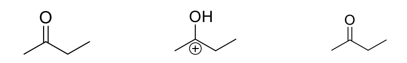			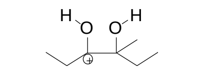	CH_3^+	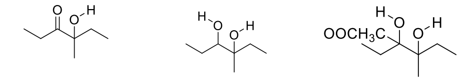	N/A
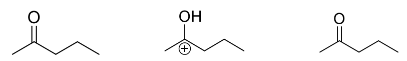			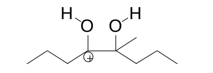	CH_3^+	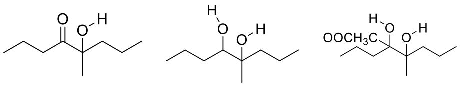	N/A
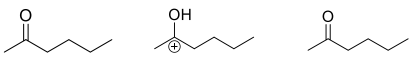			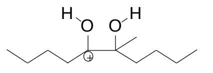	CH_3^+	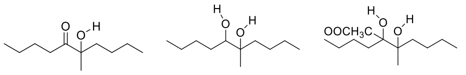	N/A
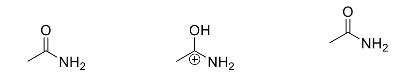			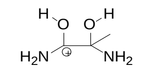	CH_3^+	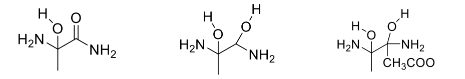	N/A
			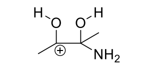	NH_2^+	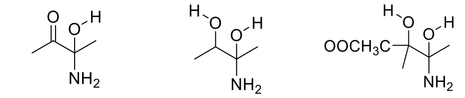	N/A
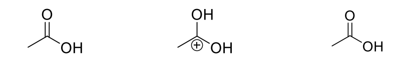			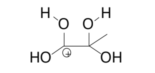	CH_3^+	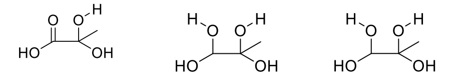	N/A
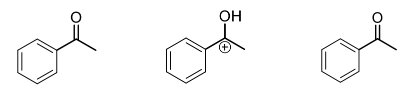			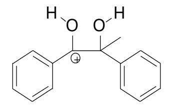	CH_3^+	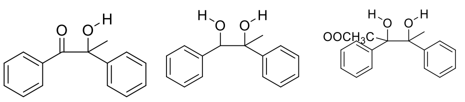	N/A
			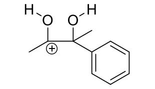	$C_7H_7^+$	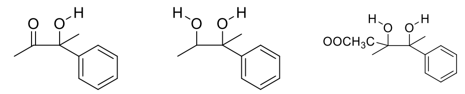	
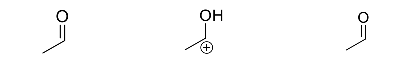			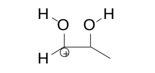	CH_3^+	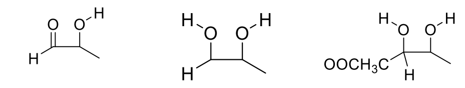	N/A
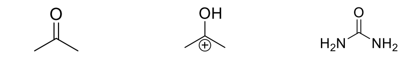			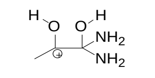	CH_3^+	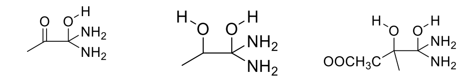	N/A
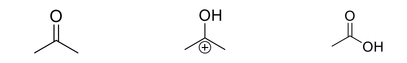			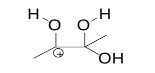	CH_3^+	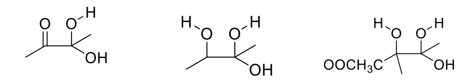	N/A
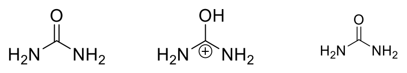			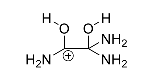	NH_2^+	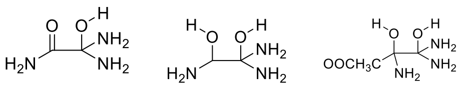	N/A
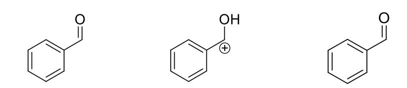			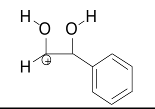	$C_7H_7^+$	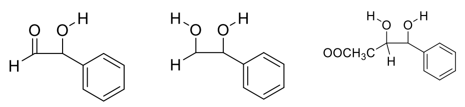	N/A

N/A: not detected.

Supplementary Table 3. [M + H]⁺, [M + Na]⁺ and the corresponding demethylation/deamination/dephenylation C–C coupling product ions of compounds containing carbonyl studied in the tandem mass spectrometry spectra in the gas phase.

Reactant a		Reactant b		C–C coupling product ions	Leaving group	Yields
Neutral form	Charged form	Neutral form	Charged form			
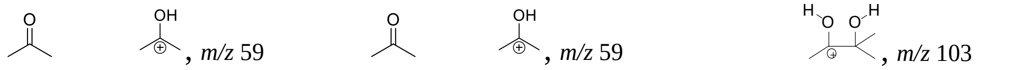	, *m/z* 59		, *m/z* 59	, *m/z* 103	CH_3^+	23%
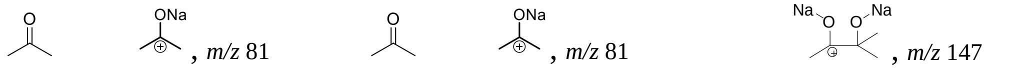	, *m/z* 81		, *m/z* 81	, *m/z* 147	CH_3^+	25%
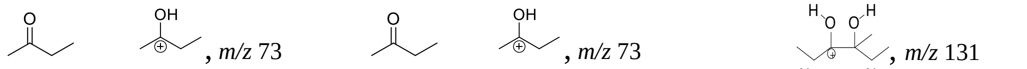	, *m/z* 73		, *m/z* 73	, *m/z* 131	CH_3^+	13%
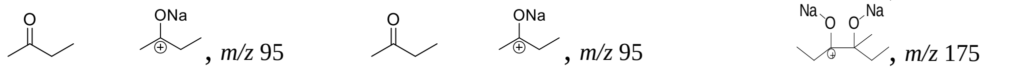	, *m/z* 95		, *m/z* 95	, *m/z* 175	CH_3^+	2%
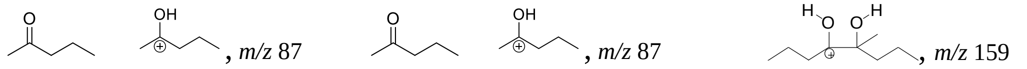	, *m/z* 87		, *m/z* 87	, *m/z* 159	CH_3^+	2%
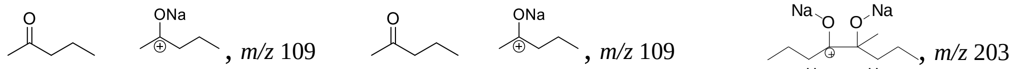	, *m/z* 109		, *m/z* 109	, *m/z* 203	CH_3^+	16%
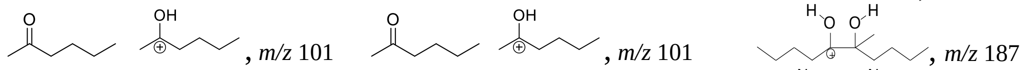	, *m/z* 101		, *m/z* 101	, *m/z* 187	CH_3^+	4%
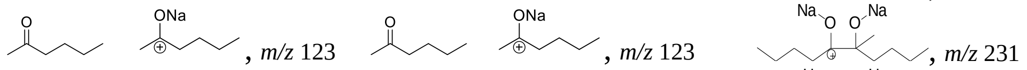	, *m/z* 123		, *m/z* 123	, *m/z* 231	CH_3^+	14%
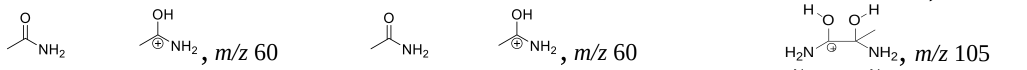	, *m/z* 60		, *m/z* 60	, *m/z* 105	CH_3^+	0.1%
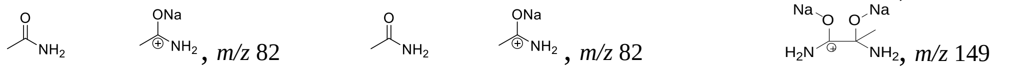	, *m/z* 82		, *m/z* 82	, *m/z* 149	CH_3^+	0.2%
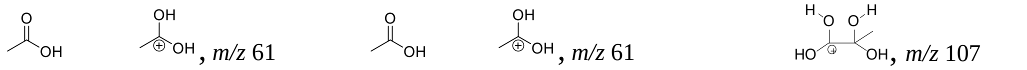	, *m/z* 61		, *m/z* 61	, *m/z* 107	CH_3^+	29%
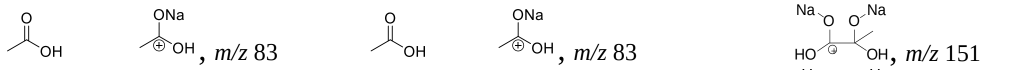	, *m/z* 83		, *m/z* 83	, *m/z* 151	CH_3^+	0.1%
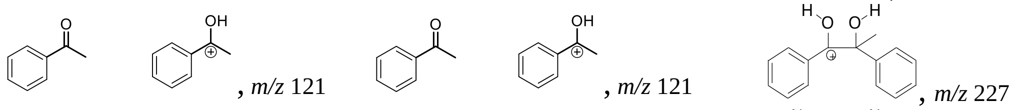	, *m/z* 121		, *m/z* 121	, *m/z* 227	$C_7H_7^+$	3%
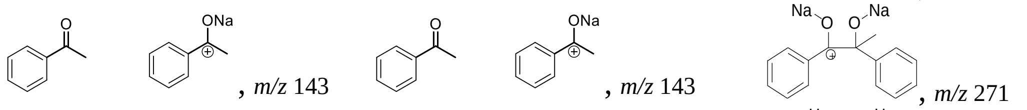	, *m/z* 143		, *m/z* 143	, *m/z* 271	$C_7H_7^+$	0.1%
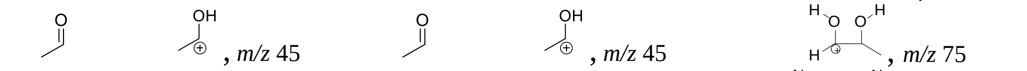	, *m/z* 45		, *m/z* 45	, *m/z* 75	CH_3^+	9%
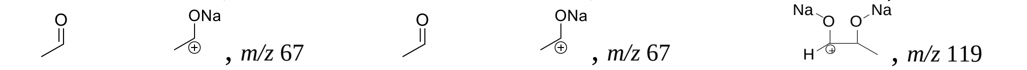	, *m/z* 67		, *m/z* 67	, *m/z* 119	CH_3^+	14%
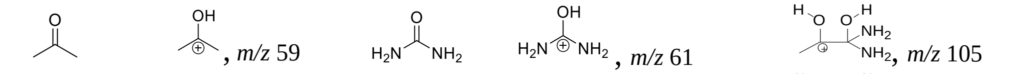	, *m/z* 59		, *m/z* 61	, *m/z* 105	CH_3^+	19%
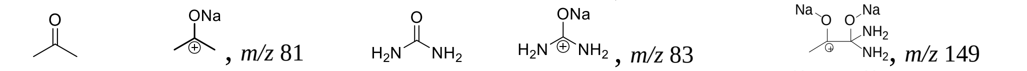	, *m/z* 81		, *m/z* 83	, *m/z* 149	CH_3^+	14%
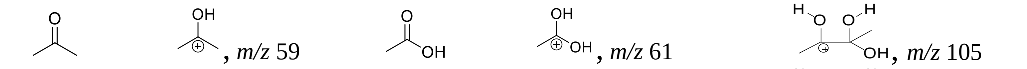	, *m/z* 59		, *m/z* 61	, *m/z* 105	CH_3^+	8%
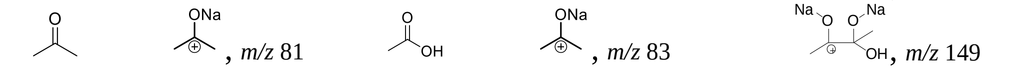	, *m/z* 81		, *m/z* 83	, *m/z* 149	CH_3^+	16%
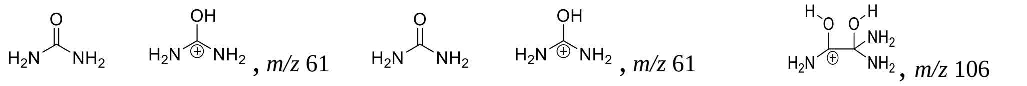	, *m/z* 61		, *m/z* 61	, *m/z* 106	NH_2^+	0.1%

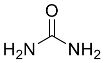	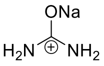, *m/z* 83	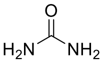	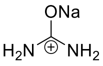, *m/z* 83	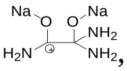, *m/z* 150	NH_2^+	0.1%
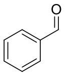	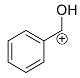, *m/z* 107	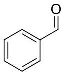	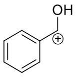, *m/z* 107	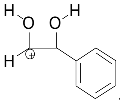, *m/z* 137	$C_7H_7^+$	68%
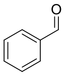	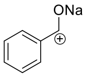, *m/z* 129	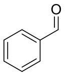	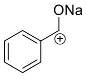, *m/z* 129	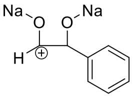, *m/z* 181	$C_7H_7^+$	54%

Note that the ion trap instrument was a commercial product. The reaction yields could be further optimized using a totally customized reactor.

The imaginary vibrational frequency of TS1 and TS2, together with the eigenvector of this vibrational mode.

TS1 Frequencies -- -1122.13

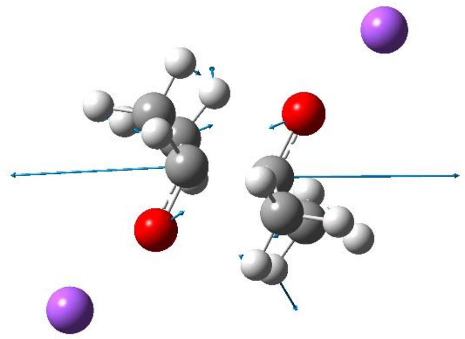

Atom	AN	X	Y	Z
1	6	-0.5	-0.36	0.1
2	8	0.12	0.02	-0.06
3	6	0.03	0.04	-0.05
4	6	0.12	0.03	0.01
5	1	-0.01	0	-0.05
6	1	-0.01	0.01	-0.02
7	1	0	0.06	-0.01
8	1	0.03	0.07	0.03
9	1	0.03	-0.12	0.2
10	1	0.02	0.07	-0.03
11	11	0.01	0.01	0
12	6	0.5	0.36	0.1
13	8	-0.12	-0.02	-0.06
14	6	-0.12	-0.03	0.01
15	6	-0.03	-0.04	-0.05
16	1	0.01	-0.01	-0.02
17	1	0.01	0	-0.05
18	1	-0.03	0.12	0.2
19	1	-0.02	-0.07	-0.03
20	1	0	-0.06	-0.01
21	1	-0.03	-0.07	0.03
22	11	-0.01	-0.01	0

TS2 Frequencies -- -261.6358

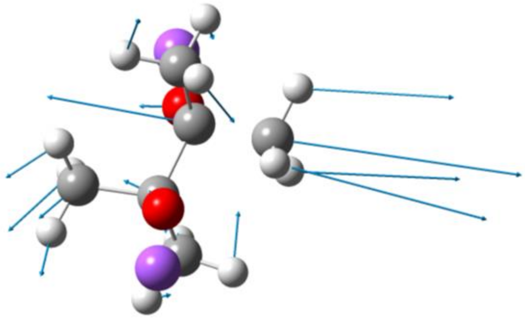

Atom	AN	X	Y	Z
1	6	0.04	-0.23	-0.21
2	8	-0.01	-0.07	-0.06
3	6	0.01	-0.02	0.00
4	6	-0.06	0.40	0.34
5	1	-0.07	0.12	-0.09
6	1	0.02	0.27	0.23
7	1	0.02	-0.03	0.06
8	1	0.02	-0.02	0.14
9	1	-0.07	0.31	0.33
10	1	-0.07	0.29	0.17
11	11	0.01	0.01	0.01
12	6	0.03	-0.05	-0.06
13	8	0.03	-0.01	-0.04
14	6	0.04	0.01	-0.03
15	6	-0.06	-0.09	-0.01
16	1	0.09	0.06	-0.07
17	1	-0.05	-0.12	-0.02
18	1	0.02	-0.02	-0.03
19	1	0.00	0.05	0.02
20	1	-0.11	-0.03	0.02
21	1	-0.10	-0.15	-0.01
22	11	0.00	0.01	0.01

Supplementary references

1. Xiong, H., Lee, J. K., Zare, R. N. & Min, W. Strong electric field observed at the interface of aqueous microdroplets. *J. Phys. Chem. Lett.* **11**, 7423-7428 (2020).
2. Doyle, C. C., Shi, Y. & Beck, T. L. The importance of the water molecular quadrupole for estimating interfacial potential shifts acting on ions near the liquid–vapor interface. *J. Phys. Chem. B* **123**, 3348-3358 (2019).
3. Cendagorta, J. R. & Ichiye, T. The surface potential of the water–vapor interface from classical simulations. *J. Phys. Chem. B* **119**, 9114-9122 (2015).
4. Leung, K. Surface potential at the air− water interface computed using density functional theory. *J. Phys. Chem. Lett.* **1**, 496-499 (2010).
5. Basuri, P., Gonzalez, L. E., Morato, N. M., Pradeep, T. & Cooks, R. G. Accelerated microdroplet synthesis of benzimidazoles by nucleophilic addition to protonated carboxylic acids. *Chem. Sci.* **11**, 12686-12694 (2020).
6. Girod, M., Moyano, E., Campbell, D. I. & Cooks, R. G. Accelerated bimolecular reactions in microdroplets studied by desorption electrospray ionization mass spectrometry. *Chem. Sci.* **2**, 501-510 (2011).
7. Nam, I., Lee, J. K., Nam, H. G. & Zare, R. N. Abiotic production of sugar phosphates and uridine ribonucleoside in aqueous microdroplets. *Proc. Natl. Acad. Sci.* **114**, 12396-12400 (2017).
8. Lee, J. K., Kim, S., Nam, H. G. & Zare, R. N. Microdroplet fusion mass spectrometry for fast reaction kinetics. *Proc. Natl. Acad. Sci.* **112**, 3898-3903 (2015).
9. Douglas, D. J., Frank, A. J. & Mao, D. Linear ion traps in mass spectrometry. *Mass Spectrom. Rev.* **24**, 1-29 (2005).
