# Supplementary material for: Demethylation C–C coupling reaction facilitated by the repulsive Coulomb force between two cations
Source: Nat Commun. 2024 Jul 13;15:5881. doi: 10.1038/s41467-024-49946-y (PMC11245495; doi:10.1038/s41467-024-49946-y)
Supplement: Supplementary file 3 — Description of Additional Supplementary Files [file 41467_2024_49946_MOESM3_ESM.pdf]

Description of Additional Supplementary Files

File name: Supplementary Movie 1
Description: An animation for the reaction via TS1
